# Sleep-Based Brain Age Is Reduced in Advanced Inner Engineering Meditators

**DOI:** 10.1007/s12671-025-02583-y

**Published:** 2025-05-16

**Authors:** Jayme C. Banks, Sepideh Hariri, Kestutis Kveraga, An Ouyang, Kaileigh Gallagher, Syed A. Quadri, Ryan A. Tesh, Preeti Upadhyay Reed, Robert J. Thomas, M. Brandon Westover, Haoqi Sun, Balachundhar Subramaniam

**Affiliations:** 1 Department of Neurology, Massachusetts General Hospital (MGH), 55 Fruit Street, Boston, MA 02114 USA; 2 Clinical Data Animation Center (CDAC), MGH, Boston, MA USA; 3 Sadhguru Center for a Conscious Planet, Department of Anesthesia, Critical Care and Pain Medicine, Beth Israel Deaconess Medical Center, Boston, MA USA; 4 Department of Anesthesia, Harvard Medical School, Boston, MA USA; 5 Division of Pulmonary, Critical Care, & Sleep, Department of Medicine, Beth Israel Deaconess Medical Center (BIDMC), Boston, MA USA

**Keywords:** Meditation, Sleep, Electroencephalography, Brain health, Wearable device

## Abstract

**Objectives:**

We aimed to quantify the effects of advanced meditation on brain electrical activity during sleep. This investigation addresses the need for objective neurophysiological measures of meditation’s potential impact on brain aging and health.

**Method:**

This study was a single-site, prospective cohort study (conducted August 25, 2021, through September 26, 2021) of meditators attending the “Samyama Sadhana” retreat (September 1–5, 2021). Two healthy comparison groups and four comparison groups with varying degrees of age-related brain pathology are included. Using overnight electroencephalography, physiological measures of brain age were derived and subtracted from chronological age, measuring the deviation of apparent brain age from chronological age.

**Results:**

Thirty-four participants completed the study (average age = 38 years; 36% female). Estimated brain age index after adjustment by matching: meditators (*n* = 34), − 5.9 years (*SE* = 0.94 years, *t*-test* p* < 0.001); Dreem healthy controls (*n* = 1077), − 0.24 (0.61, *p* < 0.001); Massachusetts General Hospital (MGH) healthy controls (*n* = 112), 0.55 (0.92, *p* < 0.05); MGH “no dementia” (*n* = 7618), 2.4 (0.094, reference cohort for *t*-test); MGH “symptomatic” (*n* = 697), 2.0 (0.33, *p* > 0.05); MGH “mild cognitive impairment (MCI)”(*n* = 205), 8.8 (2.8, *p* < 0.05); and MGH “dementia” (*n* = 153), 10.5 (2.8, *p* < 0.01).

**Conclusions:**

Long-term meditators exhibit lower brain age relative to matched control groups. This study suggests that advanced meditation enhances brain health.

**Preregistration:**

This study was not preregistered.

**Supplementary Information:**

The online version contains supplementary material available at 10.1007/s12671-025-02583-y.

Worldwide cases of dementia and age-related cognitive decline are escalating due to increased life expectancy (World Health Organization [WHO], 2017; WHO, 2018), placing a greater burden on healthcare systems. Meditation seems to foster significant improvements in brain health, enhancing cognitive function and overall mental well-being (Luders et al., 2016). Magnetic resonance imaging (MRI) studies claim beneficial effects of meditation on brain health, associated with structural changes in gray and white matter (Fox et al., 2014, 2016; Pernet et al., 2021). Mindfulness meditation may increase neural synchrony and coherence (Fox & Cahn, 2020; Van Lutterveld et al., 2017). Some researchers hypothesized these effects are related to improved sleep quality and efficiency (Martires & Zeidler, 2015; Ong et al., 2014). In a scholarly review of 3303 records, with 18 trials and 1654 participants, researchers found moderate support that mindfulness meditation improves sleep quality (Gong et al., 2016).

Previous research on sleep following a 4-day meditation retreat has demonstrated significant improvements in both sleep quality and duration up to 40 days post-retreat (Kanchibhotla et al., 2021), corroborating existing literature on the positive impact of meditation on sleep (Baecker et al., 2021; Black et al., 2015; Nagendra et al., 2012; Nanthakwang et al., 2020; Neuendorf et al., 2015; Rusch et al., 2019). Researchers have also hypothesized that advanced meditators have increased sleep efficiency, meaning they may have more restorative sleep (Nagendra et al., 2012). Overnight polysomnography (PSG) tests of Vipassana meditators demonstrate increased durations in the restorative stages of slow wave sleep and rapid eye movement (REM) sleep (Sulekha et al., 2006). Interestingly, these beneficial effects on sleep quality have been reported as long as 5 and 12 months later, and meditation retreats are considered as effective as other evidence-based sleep treatments, according to a systematic review of studies (Kanchibhotla et al., 2021; Rusch et al., 2019). Other research suggests greater theta-alpha power during Stage 3 and 4 sleep, and an increased REM density (Mason et al., 1997). Compared to beginners, experienced Vipassana meditators exhibit changes in REM sleep organization, which may be due to greater brain plasticity associated with intense meditation (Maruthai et al., 2016).

Brain age (BA) estimates the biological age of the brain, calculated by comparing measurements of brain structure or function with age norms and selecting the age that matches best (Baecker et al., 2021). Brain age index (BAI) is the difference between BA and chronologic age (CA); that is, BAI = BA − CA. BAI has been calculated using structural measurements from MRI and functional measurements of overnight sleep electroencephalography (EEG) (Sun et al., 2019). Sleep-EEG, though less commonly used, offers key advantages over MRI: it is cost-effective, can be easily conducted at home, reflects real-time brain function and electrophysiology, and allows for repeated measurements, making it a practical tool for studying brain aging.

The sleep-EEG-based brain age index from Sun et al. (2019) has been validated as a tool to measure subclinical health conditions, accurately determining the 10-year risk within a 95% confidence interval for health outcomes including ischemic stroke, intracranial hemorrhage, mild cognitive impairment, dementia, atrial fibrillation, myocardial infarction, type 2 diabetes, hypertension, bipolar disorder, depression, and mortality in a cohort of approximately 15,000 individuals (Sun et al., 2024). This index has also been validated as a tool to measure aging, showing promise as a potential biomarker for progressive brain processes that ultimately lead towards dementia (Ye et al., 2020). Using the BAI described in Sun et al. (2019), researchers have reported increased sleep-EEG-based BAI as a predictor of increased mortality (Paixao et al., 2020) that is elevated in chronic diseases affecting brain health including hypertension and diabetes (Sun et al., 2019), HIV infection (Leone et al., 2021), and neurodegenerative disease (Ye et al., 2020).

The BAI in Sun et al. (2019) has previously been reported for individuals in the Massachusetts General Hospital (MGH) clinical sleep lab with pre-existing diagnoses of dementia, mild cognitive impairment (MCI), or cognitive symptoms without an MCI or dementia diagnosis, as well as for individuals without dementia or cognitive symptoms (Ye et al., 2020), but has not been applied to meditating populations. This BAI has recently been calculated to assess whether a 12-week aerobic exercise program reduces brain aging (Ouyang et al., 2024), suggesting it can be applied to measure reductions in brain age. In contrast, long-term meditation has been associated with reduced brain aging in MRI studies. In 2016, researchers estimated that at age 50, the MRI-based BAIs of long-term meditators were 7.5 years younger than controls (Luders et al., 2016). While certain EEG changes have been identified in advanced meditators (Kaur & Singh, 2015; Rodriguez-Larios & Alaerts, 2021; Takahashi et al., 2005) and on sleep specifically (see above), the effects of meditation on the sleep-based brain age index have not been investigated. The sleep EEG–based BAI provides a unique and novel angle: it provides a holistic measure of brain aging, combining the age-dependent changes in multiple sleep EEG microstructural features. Our paper aims to fill this gap in the scientific literature on meditation.

Since sleep can be affected by daytime activities, it is reasonable to speculate that sleep-EEG is also different in meditators. The brain age measured in this study is a function of the sleep-EEG, which is also likely different in meditators. However, the exact changes in sleep EEG and brain age and their implications are yet to be explored.

Our research aimed to measure sleep BA in a cohort of advanced meditators attending the Samyama Sadhana meditation retreat compared to control populations and populations with varying degrees of brain-aging-related pathology that have previously been reported in Ye et al. (2020). Samyama Sadhana is a 4-day advanced residential meditation program that requires completion of a series of beginner to advanced yoga and meditation programs and regular daily practice, usually through a course of a few years, as well as a 40-day intensive preparation period (3–4 hr of daily practice) and specific vegan diet, leading to the retreat. This program is a refresher course for those who have previously attended an 8-day meditation program called Samyama, an advanced form of yogic meditation practice. Previous studies on those who have completed the 8-day Samyama program, which is a prerequisite for Samyama Sadhana, have shown increased acylglycines (associated with increased cellular anandamide levels, anti-inflammation, analgesia, and vascular relaxation) (Vishnubhotla et al., 2022), increased beneficial gut bacteria (Raman et al., 2023), reduced HbA1C and systemic inflammation, improved lipid profile, and enhanced mental health outcomes (Sadhasivam et al., 2021) and upregulation of 220 genes associated with the immune response (Chandran et al., 2021). Samyama participants have increased resting-state functional connectivity between the salience and default mode networks of the brain, assessed by functional MRI and correlated with improved self-reported mindfulness scores (Vishnubhotla et al., 2021).

While the literature commonly describes advanced meditators as those with 5000 to 6000 hr of meditation experience, we chose the Samyama Sadhana program as it requires extensive previous training, intense preparation, and an interview selection process for the prerequisite 8-day Samyama program. These specific requirements make this cohort of advanced meditators uniquely uniform in terms of their practice types and experience. This, along with the existing literature on measurable physiological, neural, and psychological benefits of these practices, suggests that Samyama Sadhana is a suitable intervention for this study. Nevertheless, we would like to emphasize two points. First, we do not mean to suggest that Samyama Sadhana meditation is the only suitable choice for a study of the effects of meditation on BAI. Second, while the advent of the Samyama Sadhana meditation retreat provided a unique opportunity to study BAI in a large group of advanced meditators, the years of meditation practice leading up to the retreat, rather than the 4-day retreat itself, is the key intervention investigated in this work.

Sleep EEG–based BAI was calculated according to existing methodologies in the literature (Sun et al., 2019) with BAI representing the deviation from normal aging patterns in the sleep EEG. We hypothesized sleep BAI would be lower in the meditation group compared to control populations, which would correlate with higher scores on cognition tests. We included additional populations with varying degrees of age-related cognitive decline since the BAI used in Sun et al. (2019) has been previously reported in these populations (Ye et al., 2020), serving as a validation to ensure the coherence and reliability of our results.

## Method

### Participants

This single-site prospective cohort study aimed to track sleep quality and brain health in a sample (*n* = 35) of advanced meditators within 1 week prior and 3 weeks following a silent meditation retreat. Participants scheduled to attend the “Samyama Sadhana” retreat from September 1 to 5, 2021, were contacted by researchers. Inclusion criteria were (1) 18 years or older; (2) current US resident; (3) previous experience attending the 8-day “Samyama” retreat; and (4) attending the “Samyama Sadhana” retreat. Selection for participation in the Samyama retreat requires confirmation during an interview of previous completion and strict dedication to the daily practice of numerous meditation techniques. Specifically, preparation for the Samyama retreat is an extensive process involving 3–4 hr of daily practice and maintenance of a vegan diet for at least 60 days prior to the retreat.

Similar to the Samyama retreat, the inclusion for the refresher course, the 4-day “Samyama Sadhana” meditation program, involves previous completion and daily practice of two breath-based seated yogic meditations called Kriyas (21-min Shambavi Mahamudra Kriya, and 40-min Shakti Chalana Kriya), two Hatha yoga practices involving yogic postures along with the breath (60 min, Yogasanas and Sun Salutations), conscious non-doing meditation (15 min twice a day, Shoonya Meditation), and alternate nostril breathing (30–60 min, Sukha Kriya). In preparation for the retreat, these practices and vegan diet are maintained for a minimum of 40 days leading to the Samyama Sadhana retreat. Participants in this study reported the number of days per week that they completed certain meditation practices for 40 days prior to the “Samyama Sadhana” retreat (Table 1). Participants reported no existing insomnia and were not currently taking antidepressants, benzodiazepines, neuroleptics, or opiates. One participant self-reported mild sleep apnea without the use of continuous positive airway pressure (CPAP)/bilevel positive airway pressure (BiPAP) machines as treatment. Participants provided verbal consent to participate in this research study as approved by all required research institutions. One subject withdrew from the study following the meditation retreat and was formally excluded from the study, and three subjects did not complete the survey on baseline meditation practices and demographic information, although demographics were collected during cognition testing for all participants.

**Table 1:** Days per week of meditation by practice

Meditation patterns^a^	No. (%)^b^ Meditation cohort (*n* = 32)^c^
SM Kriya^d^	
≤ 1 day/week	5 (15.6)
2–3 days/week	7 (21.9)
4–5 days/week	6 (18.8)
≥ 6 days/week	14 (43.8)
SC Kriya^d^	
2–3 days/week	5 (15.6)
4–5 days/week	7 (21.9)
≥ 6 days/week	20 (62.5)
Shoonya	
≤ 1 day/week	1 (3.1)
2–3 days/week	10 (31.2)
4–5 days/week	5 (15.6)
≥ 6 days/week	16 (50.0)
Samyama	
≤ 1 day/week	17 (53.1)
2–3 days/week	8 (25.0)
4–5 days/week	3 (9.4)
≥ 6 days/week	4 (12.5)
Samyama Sadhana	
≤ 1 day/week	18 (56.3)
2–3 days/week	8 (25.0)
4–5 days/week	5 (15.6)
≥ 6 days/week	1 (3.1)

Abbreviations: *SM Kriya*, Shambhavi Mahamudra Kriya; *SC Kriya*, Shakti Chalana Kriya

^a^Data reported as number (%) for categorical variables

^b^Percentages were rounded up and therefore may not add to 100%

^c^Data reported for all participants (*n* = 32) who completed the baseline survey

^d^Both SM and SC Kriya are breath-based yogic practices completed in a seated posture

### Procedure

#### Meditation

The 4-day Samyama Sadhana retreat consists of 10–12 hr of yoga and meditation practices each day. The main practice that is cultivated during this program is the Samyama meditation. In classical yoga, the combined practice of Dharana, Dhyana, and Samadhi is referred to as a process called Samyama. Dharana is the process of focused attention for an extended period of time. Dhyana involves intense contemplation and Samadhi involves an experience of unification (Sadhasivam et al., 2021). Participants attending the retreat learned no new practices during the retreat and continued only the same set of practices that they reported in Table 1 after completion of the program.

#### Sleep-EEG Recording

We measured the sleep-EEG of meditators using the Dreem headband (Figure S1 in the Supplementary Information), worn for up to four consecutive nights within 1 week before and within 3 weeks after the retreat. The time window for data collection before and after the retreat was selected to allow for differing travel and work schedules of participants. Specifically, participants were allowed to stay at the ashram for additional nights if they wished, which impacted when data collection could occur. We limited the window as much as possible given these constraints.

#### Cognition and Emotion Testing

Thirty-five participants completed the pre-retreat assessment and thirty-four participants completed the post-meditation assessment. Cognitive performance and emotional domains were assessed up to 1 week before and up to 3 weeks after the meditation retreat. Tests were selected from the National Institutes of Health (NIH) Toolbox Cognition and Emotion Batteries (Fox et al., 2022; Gershon et al., 2013) because the National Institute on Aging considers these batteries to be well-validated and psychometrically sound (Hodes et al., 2013). We selected components of both crystallized and fluid cognition that could be administered electronically using an iPad during a Zoom meeting. Cognitive tests took 40 to 55 min, while emotion batteries took approximately 10 min. Participants completed assessments in one sitting and were instructed to schedule testing around the same time of day before and after the retreat to reduce within-subject variability.

#### Comparison Cohorts

**Fig. 1:**
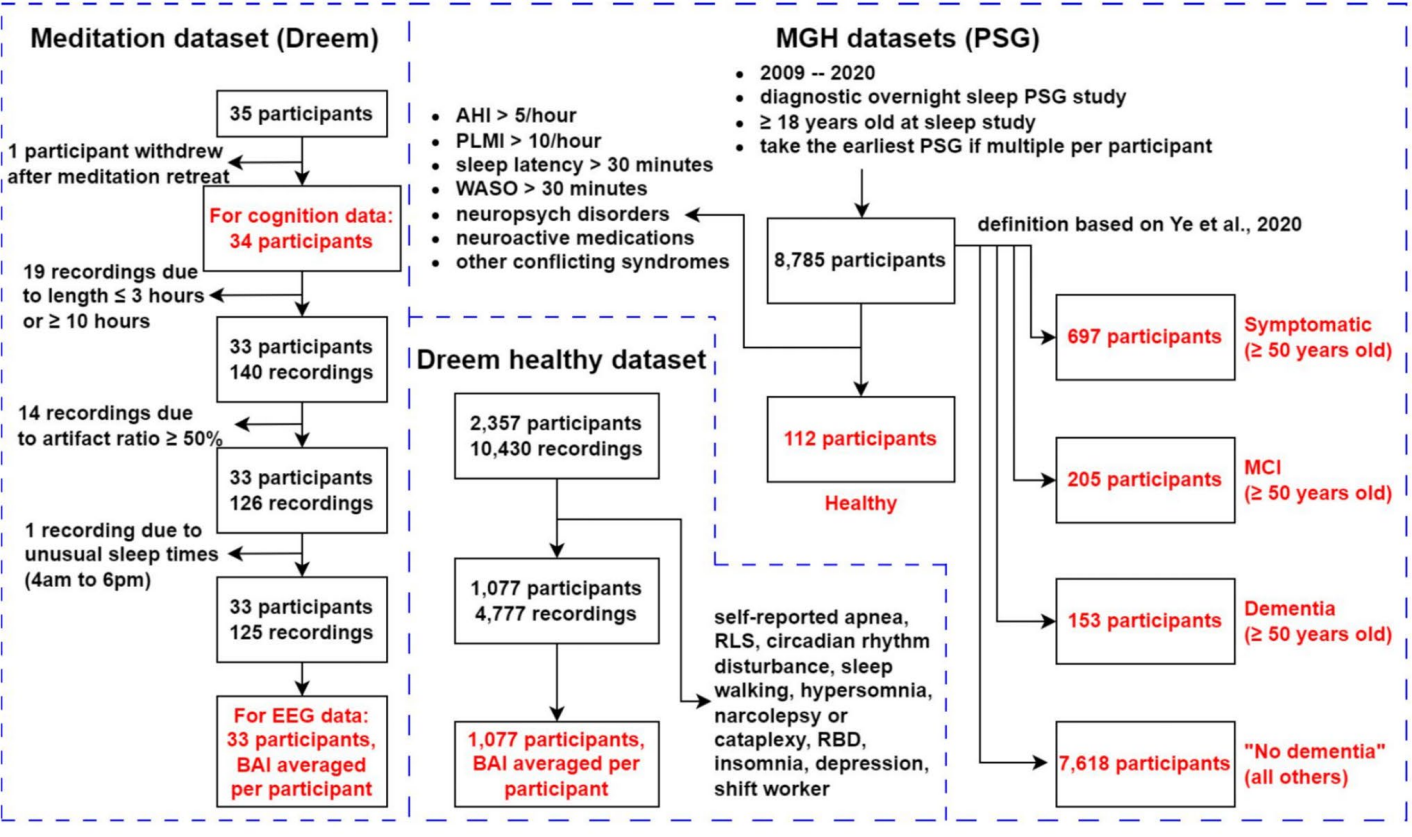
Cohort selection: flowchart showing how the final meditation cohort and other comparison cohorts are selected

We assembled six comparison groups (Fig. 1). Five comparison groups were composed of patients who underwent conventional overnight polysomnography (PSG) in a clinical sleep laboratory at Massachusetts General Hospital (MGH) equipped with the Natus PSG system, including individuals with pre-existing diagnoses of dementia (MGH “dementia”), mild cognitive impairment (MCI)(MGH “MCI”), and cognitive symptoms without an MCI or dementia diagnosis (MGH “symptomatic”), as well as for individuals without dementia or other cognitive symptoms (MGH “no dementia”) (Ye et al., 2020). Specifically, the MGH “no dementia” group can still have disease diagnoses that affect sleep-EEG, such as depression and stroke, and medications that affect sleep-EEG, such as benzodiazepines. Further descriptions of these four cohorts can be found in Ye et al. (2020). For the fifth comparison group, we restricted the MGH PSG dataset based on abnormal sleep testing results, neurological and psychiatric disorders, current use of neuroactive medications, and major health conditions to arrive at a group of healthy control subjects (MGH healthy control (HC)). Specific exclusion criteria for this cohort are provided in Table 2. Further details on the criteria used for these classifications are provided in the Supplemental Material. A final comparison cohort (Dreem healthy control), which was provided by the sleep wearables company Dreem, includes self-reported healthy individuals who underwent overnight EEG recordings using the same home-wearable device as the advanced meditation group, the Dreem headband. Sleep wearables refer to portable devices that monitor brain activity and sleep patterns, allowing for convenient data collection at home instead of in-lab settings. Exclusion criteria for this cohort are based on self-reported health and sleep metrics described in Table 2.

**Table 2:** Exclusion criteria for healthy control groups

Cohort	Details	Exclusion criteria
Dreem healthy control	Self-reported healthy controls who underwent overnight EEG recordings with the Dreem EEG device	- Apnea - Restless legs syndrome - Circadian rhythm disturbance - Sleepwalking - Hypersomnia - Narcolepsy - Cataplexy - REM-sleep behavior disorder - Insomnia - Depression - Shift worker
MGH healthy control	Healthy participants who underwent clinical polysomnography (PSG) testing in the MGH sleep lab	**Abnormal sleep testing results:** - AHI > 5 - PLMI > 10 - Sleep latency > 30 min - WASO > 30 min **Neurological and psychiatric disorders:** - Bipolar disorder - Alcohol abuse - Encephalopathy - Depressive disorders - Huntington’s chorea - Stroke - Dementia - MCI **Neuroactive medications:** - Benzodiazepines - Antidepressants - Neuroleptics - Opiates **Major health conditions:** - Cancer - HIV - Trisomy 21

Abbreviations: *EEG*, electroencephalogram; *REM*, rapid eye movement; *MGH*, Massachusetts General Hospital; *AHI*, apnea–hypopnea index; *PLMI*, periodic limb movement index; *WASO*, wakefulness after sleep onset; *MCI*, mild cognitive impairment, *HIV*, human immunodeficiency virus

### Measures

#### Cognition and Emotion Batteries

From the NIH Toolbox Cognition Battery (Gershon et al., 2013), the tests administered were the Picture Vocabulary Test (PVT), Oral Reading Recall Test (ORRT), List Sort Working Memory Test (LSWMT), and Picture Sequence Memory Test (PSMT). These tests examined the domains of language (PVT, ORRT), working memory (LSWMT), and episodic memory (PSMT). Two versions of each of these tasks were administered at the different time points to remove practice effects. Three tests from the Cognition Battery that test executive function (Dimensional Change Card Sort Test), attention (Flanker Inhibitory Control and Attention Test), and processing speed (Pattern Comparison Processing Speed Test) were not tested as they were unable to be administered over Zoom. The NIH Toolbox Emotion Battery (Gershon et al., 2013) involved seven 2-min assessments: Positive Affect, Life Satisfaction, Emotional Support, Instrumental Support, Friendship, Loneliness, and Perceived Stress. Emotional Support evaluates the participant’s perception of having empathetic, caring individuals in their social network who are available to listen. Friendship measures the availability of friends or companions for interaction. General Life Satisfaction assesses whether participants enjoy and find contentment in their life experiences. Instrumental Support evaluates perceptions of material or functional aid being available for daily tasks. Loneliness measures feelings of social isolation or being alone. Perceived Stress assesses how unpredictable, uncontrollable, and overwhelming participants find their lives. Positive Affect evaluates feelings of happiness, excitement, and contentment in the environment.

#### Sleep-EEG-Recordings

For pre-retreat sleep EEGs, two participants had four nights, fourteen had three nights, seven had two nights, eight had one night, and three had none. For post-retreat sleep EEGs, five participants had four nights, fifteen had three nights, seven had two nights, three had one night, and four had none. Recordings shorter than 3 hr or longer than 10 hr, containing more than 50% artifact, or at unusual sleep times (4:00 am to 6:00 pm) were excluded from analysis (Fig. 1). The duration of sleep EEG recordings was variable since participants wore the Dreem EEG headbands at home and followed their habitual sleep times. Each participant from the Dreem healthy control cohort recorded multiple nights of EEG (6 on average) using the Dreem EEG device.

#### Exposure

There are two different exposures on the study cohort: (1) long-term advanced meditation practice and (2) participation in the meditation retreat.

#### Outcomes

We evaluated two outcomes in the advanced meditators: (1) Cognition and Emotion Battery performance, and (2) sleep EEG–based brain age index (BAI).

#### Confounding Factors

Several potential confounding factors were pre-specified, with a focus on the covariates to be adjusted for in the analysis. Only those covariates that affected both the exposure (meditation) and the outcome (brain age index) were classified as confounding variables. These confounding factors included age, sex (male/female), education level (high school/college/graduate), and race (Asian, Black or African American, Multi-Racial, White, or Other). Given that the meditation dataset comprised solely participants identifying as non-Hispanic, ethnicity was not treated as a confounding variable but was instead included as a covariate. Additionally, we measured as a confounding variable the percentage of individuals who were bilingual, but spoke English as a second language, given the large percent of individuals in the cohort who were fluent in English but immigrated from India. These confounding factors were measured for both the meditation cohort and the MGH comparison cohorts. For the Dreem healthy controls, only age and sex information were available.

### Data Analyses

#### Cognition and Emotion Testing

To analyze Cognition Battery results, we used corrected T-scores (adjusted for the key demographic variables of age, gender, race/ethnicity, and education level) generated by the NIH Toolbox iPad app for analysis. Repeated measures ANOVAs performed using JASP software version 0.16.3 were used to assess changes in adjusted measures. Cognitive and emotional scores were analyzed separately.

#### EEG Preprocessing and Artifact Removal

We employed standard filtering and artifact removal approaches. For the Dreem device signals (meditation cohort, Dreem healthy control cohort), we used two bipolar EEG channels: F7-O1 and F8-O2. The sampling rate was 250 Hz, i.e., 250 sampling points per second. EEG signals were notch-filtered at 60 Hz to reduce background noise and bandpass-filtered from 0.5 to 20 Hz to reduce myogenic artifacts. Signals were then segmented into 30-s epochs.

For the Dreem device signals, epochs with artifacts were excluded in two steps. In the first step, we removed “definite” artifacts by excluding epochs with a maximum absolute amplitude larger than 500 µV or a low (standard deviation < 1 µV) amplitude that lasted 2 s or longer. In the second step, for the remaining epochs, we applied a trained linear discriminant analysis (LDA) classifier to classify each epoch as artifact vs. not artifact. The details are described in the Supplementary Method.

For clinical PSGs (MGH cohorts), we used six EEG channels, including F3-M2, F4-M1, C3-M2, C4-M1, O1-M2, and O2-M1. The sampling rate was 200 Hz. Signals were preprocessed in the same way as the Dreem EEG signals. For artifact removal, we removed the “definite” artifacts as defined in the first step above, since the clinical-grade PSG signals are cleaner than the signals from the Dreem devices used at home.

#### EEG Spectrum and Spectrogram

We used the multitaper method (Prerau et al., 2017; Thomson, 1982) to estimate the power spectral density for each 30-s epoch of the EEG during sleep. We used 18 tapers, which produced a half-bandwidth of 0.67 Hz. Spectrograms were generated by a sequence of spectra estimated using 30-s non-overlapping epochs. For visualization, we limited spectrogram frequencies from 0.5 to 20 Hz, and then converted the units to decibels (dB).

To further minimize potential non-physiological differences between EEGs from the Dreem device and clinical PSGs from the MGH comparison cohorts, we applied spectral normalization (Grandchamp & Delorme, 2011) to bring power spectral densities (PSD) of Dreem EEG recordings at the group level into closer alignment with appropriately matched PSG recordings from the reference group (MGH “no-dementia” group). For each sleep stage, age group, sex, and electrode channel, we computed the average PSD across all available 30-s epochs for both Dreem and MGH PSG EEGs from the reference group. The inverse Fourier transform of the ratio between the average PSD in the MGH “no dementia” group to the Dreem group (PSG/Dreem) provides the desired temporal filtering kernel. The rationale is the convolution theorem, where the Fourier transform of a convolution of two signals is the product of their Fourier transforms. We therefore filtered Dreem EEGs with this filtering kernel to perform spectral normalization. We acknowledge that some meditation-related differences could be under-estimated as a result of filtering.

#### Effects of Advanced Meditation-Based Brain Age Index (BAI)

Brain age index (BAI) is the difference between brain age (BA) and chronological age (CA): BAI = BA − CA. BA is calculated by first extracting 102 features from each 30-s epoch covering both time and frequency domains as described in Sun et al., 2019, then taking the average of these features across epochs from the same sleep stage, forming 102 × 5 = 510 features per EEG, and finally combining these features as a weighted sum. The frequency-domain features include statistics of delta (1–4 Hz), theta (4–8 Hz), alpha (8–12 Hz) band power, and power ratios. The time-domain features include statistics and entropy of the signals. We derived the weights from a model trained on 2,330 participants identified as “brain healthy,” meaning they had not been diagnosed with any significant neurological or psychiatric disorders at the time of the polysomnography (PSG) (Paixao et al., 2020).

The EEG channels from the Dreem device differ from the PSGs which were initially used to train the BAI model. To obtain the BAI for Dreem data, we adapted the original BAI model by retraining it on brain-healthy participants (age range 18 to 80 years old) from a prior BAI study (Sun et al., 2019) using the F3-M2 and F4-M1 channels to approximately match the F7-O1 and F8-O2 channels in Dreem (F3 is near the location of F7, and F4 is near the location of F8). For both the meditation cohort and the Dreem healthy control cohort, we estimated the BAI by calculating the average BAI over the recording nights, so that the point estimate of BAI is comparable for participants with different numbers of nights available. Previous research using the sleep-EEG-based BAI from Sun et al. (2019) demonstrates that night-to-night variability of BAI is reduced by averaging across multiple nights (Hogan et al., 2021).

#### Effect Estimation

To estimate the effects of the retreat in the meditating cohort, we calculated the difference in scores for the Cognitive and Emotion Batteries and for the BAI before the retreat compared to after the retreat. To estimate the effects of long-term advanced meditation, we compared pre-retreat Cognitive and Emotion Battery results with published norms for the general population; and pre-retreat BAI with BAI in several non-meditator comparison groups using matching. The purpose of matching is to ensure that a comparison of the outcomes in different exposure groups is not confounded by differences in confounding factors, including age, sex, education level, and race. We used full matching from the “matchit” package in R, where every participant receives a weight so that the distribution of covariates in the weighted sample across groups is as similar as possible (Ho et al., 2007; Stuart, 2010; Thoemmes & Kim, 2011). These weights are then used in a linear regression to predict outcomes. Confidence intervals were derived using clustered covariance matrix estimation. We considered three options to serve as the reference group for matching (MGH “no dementia” group; MGH healthy control group; Dreem healthy control group). Among these, we selected the MGH “no dementia” cohort as it was the largest of the three potential reference groups.

#### Statistical Analysis

BAI for groups is summarized by group means and standard errors. Cognitive and emotional scores for the groups are summarized using means and standard deviations, as well as medians and interquartile ranges. The outcomes before and after meditation are compared using paired *t*-tests for two related samples. The outcomes with and without long-term advanced meditation practice are compared using *t*-tests with independent samples. Significance was set at *p* < 0.05/50 = 0.001, based on Bonferroni correction, where 50 is a conservative estimate of the number of tests. We define 0.001 < *p* < 0.05 as trend-level. We also analyzed years of meditation in relation to BAI to test for a longitudinal effect of meditation. Finally, we calculated the Spearman’s correlation between pre-retreat BAI versus corrected t-score of the crystallized cognition score as well as pre-retreat BAI versus Positive Affect from the Emotion Battery to investigate how well the relationship between these two variables can be described using a monotonic function, where a value of + 1 represents a perfect positive correlation and − 1 represents a perfect negative correlation.

## Results

### Cohort Characteristics

Participants’ demographics and comorbidities (Table 3) were recorded before the retreat. Thirty-four participants (average age = 38 years; 36% female) completed the study, as one person withdrew post-retreat due to a medical issue unrelated to the study. It is noteworthy that 53.1% of the meditation cohort reported being foreign-born bilingual individuals from India, with English as their second language. Additionally, 62.5% of the meditators reported a high level of education, having completed an advanced degree such as an associate’s, master’s, or doctoral degree. For comparison groups: there were 1077 Dreem healthy controls (average age = 46 years, 18% female); 112 MGH healthy controls (39 years, 55% female); 7618 in the MGH “no dementia” group (49 years, 50% female); 697 MGH “symptomatic” (64 years, 56% female); 205 MGH “MCI” (68 years, 42% female); and 153 in the MGH “dementia” group (70 years, 45% female). In Figure S2 (in the Supplementary Information), we show typical spectrograms for each group for visual comparison (in the Supplementary Information).

**Table 3:** Demographic and comorbidity for meditation cohort

Baseline characteristic^a^	No. (%)^b^
Demographic	
Female	13 (37.1)
Age, median (*IQR*)	35 (31–42)
Ethnicity	
Not Hispanic	35 (100.0)
Race^c^	
Asian	30 (85.7)
Black or African American	1 (2.9)
Multi-racial	1 (2.9)
White	3 (8.6)
Education^c^	
High school graduate	1 (2.9)
Associate degree	1 (2.9)
Bachelor’s degree	14 (40.0)
Master’s degree	16 (45.7)
Doctoral degree	3 (8.6)
Language^c^,^d^	
Foreign-born English speaker	10 (31.2)
Foreign-born^e^	17 (53.1)
Native-born English speaker	5 (15.6)
Employment status^c^,^d^	
Employed full-time	23 (71.9)
Employed part-time	1 (3.1)
Other	2 (6.3)
Self-employed	6 (18.8)
Work^c^,^f^	
20–40 hr/week	8 (25.8)
> 40 hr/week	23 (74.2)
Comorbidities	
None reported	20 (62.5)
Thyroid disease	4 (12.5)
Asthma	3 (9.4)
Gastrointestinal disease	3 (9.4)
Hypertension	2 (6.3)
Anemia	1 (3.1)
Anxiety	1 (3.1)
Rheumatoid arthritis	1 (3.1)
Back or neck pain	1 (3.1)
Diabetes	1 (3.1)
Headaches	1 (3.1)
Peripheral neuropathy	1 (3.1)
Sinusitis	1 (3.1)
COVID-19	1 (3.1)

Abbreviations: *IQR*, interquartile range; *COVID-19*, coronavirus disease 2019

^a^Data reported as number (%) for categorical variables and median (*IQR*) for continuous variables

^b^Data reported for all enrolled participants (*n* = 35) unless otherwise noted

^c^Percentages were rounded up and therefore may not add to 100%

^d^Data reported for all participants (*n* = 32) who completed the baseline survey

^e^Foreign-born with English as a second language

^f^Data reported for most participants (*n* = 31) who completed the baseline survey; one person did not complete this section of the survey

### Effects of Advanced Meditation on Brain Age Index

**Fig. 2:**
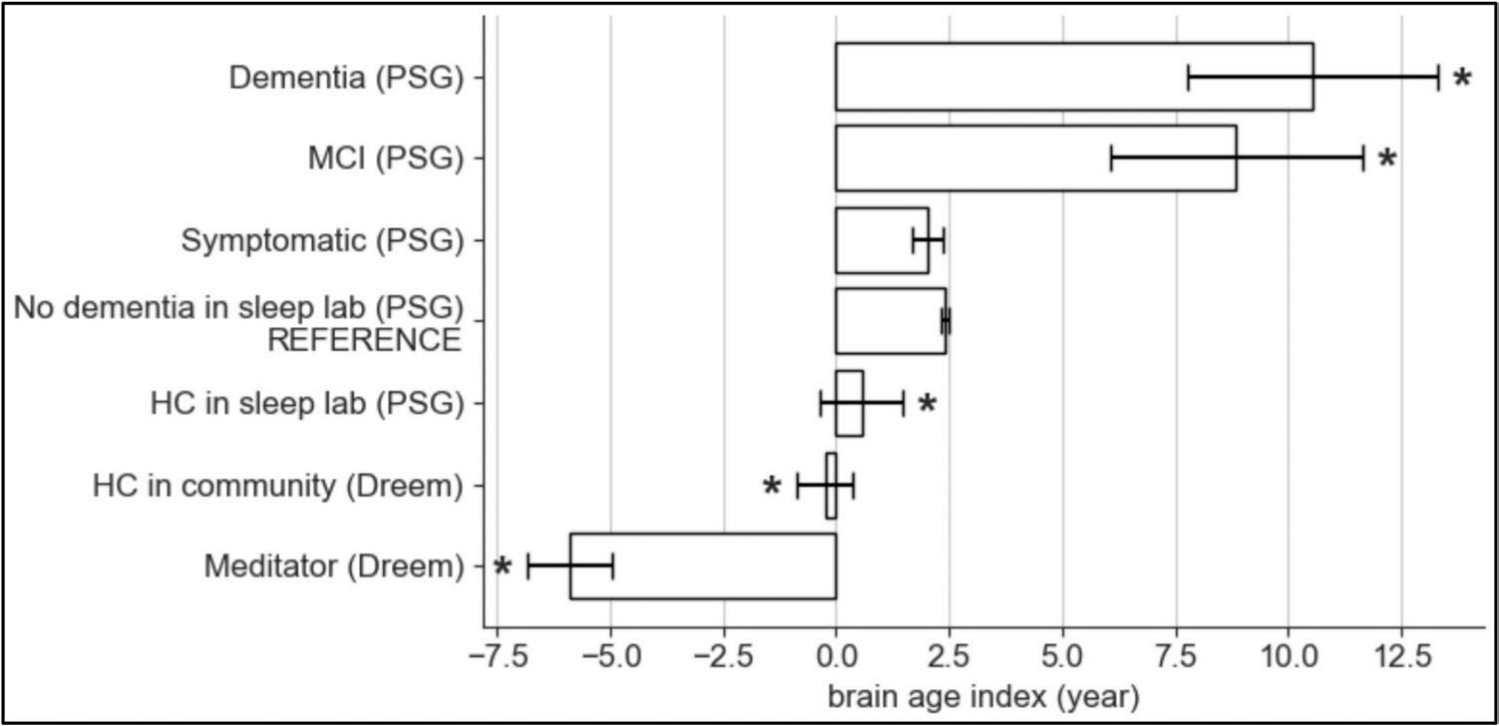
BAI by group: The brain age index (BAI) (mean, standard error) in meditators with long-term advanced meditation practice (bottom group), healthy controls (HC) from the community, HC from the Massachusetts General Hospital (MGH) sleep lab, all MGH sleep lab participants excluding people with a diagnosis of dementia, mild cognitive impairment (MCI), or symptomatic of brain decline, as well as different stages of brain decline (symptomatic, MCI, dementia) from the MGH clinical sleep lab. The BAI is expressed in the unit of years, indicating the deviation of brain age from chronological age. The mean and standard errors are based on matching to the MGH “no dementia” sleep lab group. Stars indicate statistical significance (*p* < 0.001)

We compared BAI in advanced meditators to the sub-cohorts to estimate the effects of advanced meditation (Fig. 2). The estimated BAIs after adjustment by matching are: meditation group, − 5.9 years (*SE* = 0.94 years, *p* < 0.001); Dreem healthy controls, − 0.24 (0.61, *p* < 0.001); MGH healthy controls, 0.55 (0.92, *p* < 0.05); MGH “no dementia”, 2.4 (0.094, reference cohort for *t*-test); MGH “symptomatic”, 2.0 (0.33, *p* > 0.05); MGH “MCI”, 8.8 (2.8, *p* < 0.05); and MGH “dementia”, 10.5 (2.8,* p* > 0.01). We also analyzed which sleep features (components of BAI) differed between groups (Figure S3 in the Supplementary Information). The top three EEG features that lead to younger brain age in the meditators are (1) higher frontal amplitude kurtosis during N2, which measures abrupt high-amplitude bursts likely due to K-complex; (2) higher frontal theta power kurtosis during N2, which measures the extent of transient high-power burst; and (3) higher frontal alpha power kurtosis during N2. Unadjusted estimates of BAI by group are reported as a supplement (Table S1 in the Supplementary Information).

**Fig. 3:**
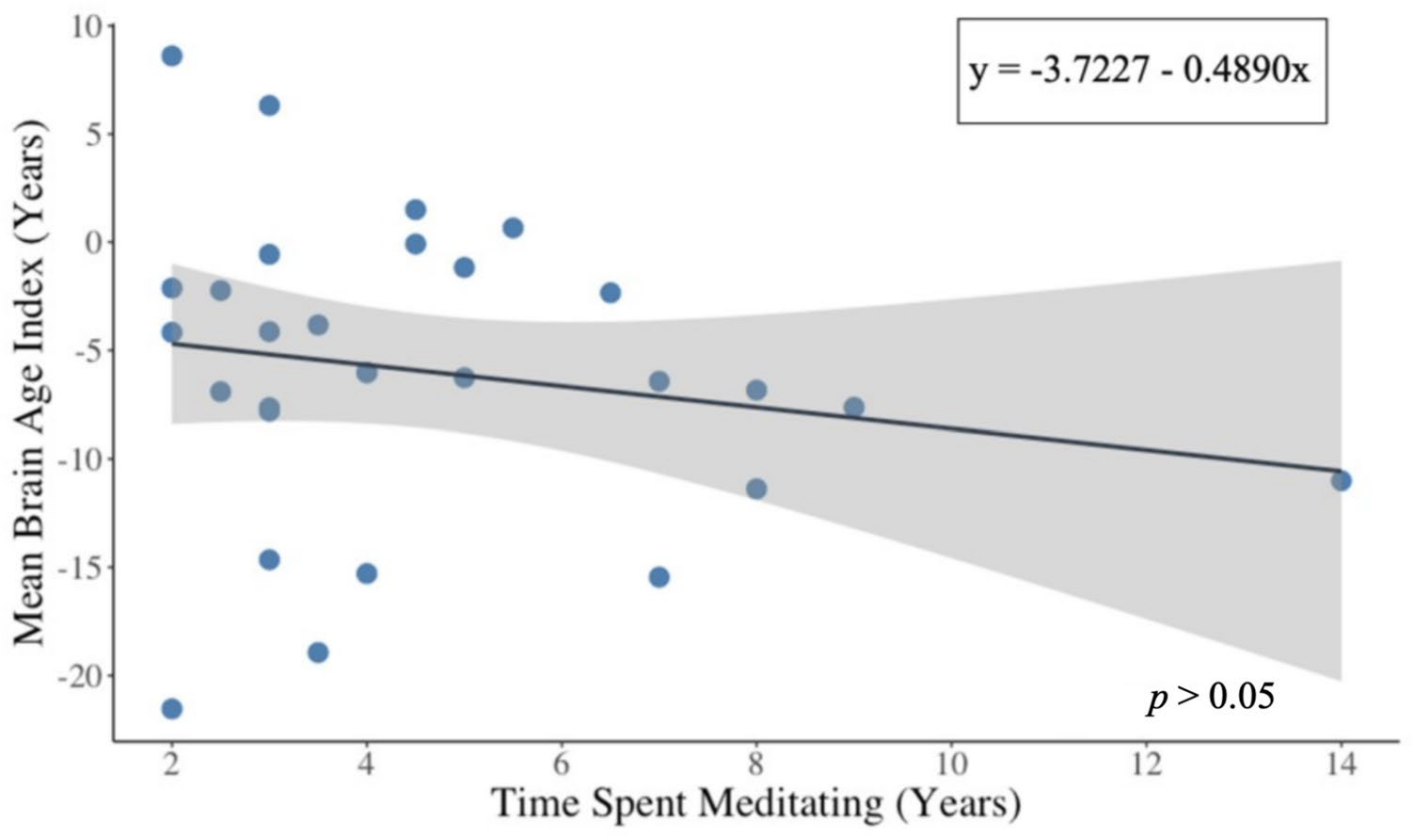
BAI vs. years of meditation: Scatter plot of years of meditation versus mean BAI for 29 participants. The black line is the least square fit to the data points. The shaded area represents the 95% confidence interval for the least square fit

We investigated whether BAI correlates with the number of years of advanced meditation practice (Fig. 3). Data were available from 29 participants; 3 meditators did not report the number of years; 2 meditators had no suitable recordings for analysis after artifact removal. The slope of the line is −0.49 years; however, this trend did not reach statistical significance (*p* > 0.05). This suggests that there is not a longitudinal effect of years of meditation on brain age index. In terms of macro-sleep characteristics, the only finding is that the mean sleep duration for the meditation cohort (6.0 hr, *SD* = 1.2 hr) was significantly lower (*p* < 0.001) than the Dreem healthy controls (7.6 hr, *SD* = 1.2 hr). We did not compare to the MGH cohorts due to the enforced wake-up time in the sleep clinic.

### Effects of the Retreat on Brain Age Index

We next compared BAI before vs. after the retreat for participants with eligible EEGs (138 of 159 recordings) and found no significant differences in group average BAI (before: − 5.9 ± 8.2 years; after − 5.0 ± 6.9 years, *p* > 0.05). Mean sleep duration before was 6.1 hr (*SD* = 1.2 hr), and after the retreat was 5.9 h (*SD* = 1.3 hr); these were not significantly different (*p* > 0.05).

### Effects of Advanced Meditation on Cognition and Emotion

We first examined how meditators compared to national norms, defined as the mean of 50 fully corrected T-scores generated by the NIH Toolbox (Table 4); both raw scores and fully corrected T-scores were produced by the NIH Toolbox and were not analyzed by our research team. Fluid cognition score means on the List Sorting Working Memory Test and the Picture Sequence Memory Test were significantly above the standardized mean of 50 (LSWMT, *t*_33_ = 3.45, *p* < 0.01; PSMT, *t*_33_ = 6.44, *p* < 0.001). For crystallized cognition, participants scored significantly higher than the national norm for the Oral Reading Recall Test (ORRT, *t*_33_ = 9.76, *p* < 0.001), but below the norm for the Picture Vocabulary Test (PVT, *t*_33_ =  − 4.01, *p* < 0.001).

**Table 4:** Raw and adjusted scores on the NIH Toolbox for meditation cohort

Assessment^a^	Raw score, mean (*SD*), median (*IQR*) (*n* = 69)^b^	Adjusted score, mean (*SD*), median (*IQR*) (*n* = 69)^b^
Cognition batteries		
Crystallized composite^c^	110.794 (6.873), 111 (10)	54.897 (8.634), 55 (12)
LSWMT	20.074 (3.092), 20 (4)	55.515 (10.747), 53 (16.25)
ORRT	116.926 (7.468), 118 (9.75)	64.221 (10.865), 66.5 (15)
PSMT	20.309 (7.819), 20 (12)	60.162 (11.931), 57 (16.75)
PVT	104.044 (8.49), 103.5 (12.25)	44.691 (9.136), 41.5 (15)
Emotion batteries		
Emotional Support	31.676 (7.214), 33.5 (11)	45.206 (10.587), 45.5 (16.25)
Friendship	28.735 (6.459), 29 (7)	46.353 (9.196), 46 (9.25)
General Life Satisfaction	27.971 (13.346), 23 (7)	57.265 (9.552), 57 (9.5)
Instrumental Support	32.529 (6.302), 33 (9)	49.441 (8.589), 48.5 (10)
Loneliness	8.618 (3.332), 8 (5.25)	49.206 (9.458), 49 (18.25)
Perceived Stress	20.588 (5.948), 21.5 (9)	43.75 (10.414), 46.5 (13.5)
Positive Affect	32.721 (21.167), 18 (41.25)	52.544 (9.302), 50 (9)

Abbreviations: *NIH*, National Institutes of Health; *IQR*, interquartile range; *LSWMT*, List Sorting Working Memory Test; *ORRT*, Oral Reading Recall Test; *PSMT*, Picture Sequence Memory Test; *PVT*, Picture Vocabulary Test

^a^Data reported as mean (*SD*), and median (*IQR*) for continuous variables

^b^Data reported for all enrolled participants (*n* = 35) for the first assessment and 34 (97%) participants for the second assessment, totaling 69 assessments

^c^Crystallized composite score is a calculation provided by the NIH Toolbox that summarizes crystallized cognition test scores. Three fluid cognition tests were omitted as they could not be completed over Zoom, so no composite fluid cognition score was generated

### Effects of the Retreat on Cognition and Emotion

Means (*SD*) and medians (*IQR*) for each time point (pre, post) are reported as supplements (Table S2 in the Supplementary Information). While measures of crystallized cognition, representing hold abilities, did not improve from pre- to post-retreat—and even declined for the ORRT—tests of fluid cognition (PSMT, LSWMT) demonstrated non-significant but trend-level improvements following the retreat. Surveys of affective measures increased significantly for positive affect and decreased non-significant but trend-level for negative affect (see Supplemental Analysis 1 in Online Resource 1).

### Correlation Between BAI vs. Cognitive and Emotion Scores in the Meditation Cohort

At the pre-retreat stage, the corrected T-score of Crystalized Cognition had the strongest Spearman’s correlation with BAI among cognitive scores, but was not significant (correlation = 0.24, *p* > 0.05). Positive Affect had the strongest Spearman’s correlation with BAI among emotion scores, but was not significant (correlation = 0.28, *p* > 0.05). At the post-retreat stage, the List Sorting Working Memory Test had the strongest Spearman’s correlation with BAI among cognitive scores, and was significantly negative (correlation =  − 0.47, *p* < 0.01), suggesting an older BAI to be associated with worse working memory. The Perceived Stress had the strongest Spearman’s correlation with BAI among emotion scores, and was significantly positive (correlation = 0.41, *p* < 0.05), suggesting an older BAI to be associated with a higher stress level. On the other hand, education level was not associated with either pre-retreat and post-retreat BAIs.

## Discussion

The growing body of literature is pointing to the impact of meditation on brain age, making meditation a very attractive non-pharmacological potential intervention for age-related cognitive decline and neurodegenerative conditions. Aging impacts the brain structure, function and biochemistry. The underlying mechanisms leading to the age-protective effects of meditation on the brain are not well understood (Kurth et al., 2017). Some of the causal pathways may be related to slower cellular aging (Gomutbutra et al., 2020; Schutte et al., 2020), reduced inflammaging (Creswell et al., 2012; Creswell et al., 2016; Malarkey, Jarjoura, & Klatt, 2013; Ng et al., 2020; Rao et al., 2015; Sadhasivam et al., 2021), increased blood biomarkers associated with development, plasticity and longevity of neuronal cells (De Melo Reis et al., 2021; Sadhasivam et al., 2020), and enhanced emotional regulation and mental well-being which in turn can lead to a more balanced homeostatic state (Gál et al., 2021; Khoury et al., 2017; Kurth et al., 2017).

Regional volumetric brain atrophy and loss of cortical thickness are the known effects of normal aging on the brain. Meditation is associated with a slower decline or even increase in cortical thickness and gray matter volume in several areas of the brain (Afonso et al., 2020; Fox et al., 2014; Kurth et al., 2023; Pernet et al., 2021), as well as improved white matter connectivity (Laneri et al., 2016; Perez-Diaz et al., 2024) as depicted by MRI studies.

Brain functional connectivity is altered across the life span (Baghernezhad & Daliri, 2024; Farras-Permanyer et al., 2019) and brain age can be estimated with machine learning algorithms using fMRI resting state functional connectivity data (Lund et al., 2022; Millar et al., 2022; Sone & Beheshti, 2022). Meditation is known to alter the brain’s functional connectivity (Rathore et al., 2022; Vishnubhotla et al., 2021). However, to our knowledge, there is no published study estimating brain age in meditators compared to controls, using functional MRI modality.

Brain electrical activity is also altered with age. Several studies suggest that the Gamma band power is negatively correlated with age (Magalhaes et al., 2018; Scott et al., 2015) while others suggest the opposite correlation (Al Zoubi et al., 2018; Gao et al., 2017), potentially indicating un-accounted various mental and emotional state of the subjects. There is a sparsity in literature on the impact of meditation on brain age using EEG data. A randomized controlled study on middle-aged adults showed increased fast wave (beta and gamma) resting state power after 7 weeks of meditation practice suggesting the reversal of the age-mediated change in brain electrical activity with meditation (Lee et al., 2024).

Our results suggest that meditator brains are on average 5.9 years younger than matched healthy controls. These results agree with previously reported data showing 7.5 years younger brains in long-term meditators of age 50, compared to controls using structural MRI pattern recognition (Luders et al., 2016).

Human sleep shows significant, predictable changes with aging, both in sleep architecture and EEG patterns. Older adults tend to sleep and wake earlier, experience shorter sleep durations, more fragmented sleep, and reduced REM and deep NREM sleep (Mander et al., 2017; Scullin, 2017; Sun et al., 2018). At the EEG level, older people exhibit fewer slow waves, decreased sleep spindle activity, and less synchronization between slow oscillations and spindles during deep sleep (Carrier et al., 2001; Helfrich et al., 2017; Larsen et al., 1995; Purcell et al., 2017; Sun et al., 2019). Older BAI is associated with advanced dementia (Ye et al., 2020), higher mortality (Paixao et al., 2020), lower crystallized cognition (Adra et al., 2023), HIV infection (Leone et al., 2021), and epilepsy (Hadar et al., 2024), while younger BAI is associated with exercise (Ouyang et al., 2024) and CPAP treatment (Yook et al., 2024).

Long-term advanced meditation practice was associated with decreased sleep-EEG-based brain age. The meditation cohort also performed significantly better than standardized norms across several cognitive domains, including both tests of fluid cognition and the crystallized cognition composite score. Measures of crystallized cognition (besides the ORRT) did not change significantly before versus after the retreat, offering support that these static measures accurately describe our meditation cohort. We did not find evidence that the retreat measurably affected BAI; we believe these physiological changes develop over longer periods. This hypothesis is supported by research applying the BAI in Sun et al. (2019) to a cohort completing a 12-week exercise program, in which cognitive scores on the NIH Toolbox improved but changes in BAI and sleep architecture were not significant (Ouyang et al., 2024). Higher scores on cognitive tests compared to the general population may be due to long-term meditation practice as opposed to meditation retreat attendance. Instead, the benefits of retreat participation were evident in Emotion Battery results, namely increased perceptions of positive affect, friendship, and emotional support, alongside decreased stress and loneliness. Overall, these results are consistent with the hypothesis that advanced meditation improves brain health over time, although it suggests that these effects are not significant enough to measure over the course of a brief meditation retreat.

Given the importance of sleep in brain health, one possible interpretation of our findings is that long-term meditation practice improves sleep in some way, and that this improves brain health. However, if so, our results are more consistent with the hypothesis that meditation improves sleep quality, as opposed to sleep duration. While some studies found improved sleep durations in meditators, other researchers have attempted to investigate an old Buddhist claim that proficient meditators sleep less than the normal population. Buddhist texts assert that meditators need only 4 hr of sleep per night, and that reduced sleep time is indicative of proficiency in meditative practices (Britton et al., 2014). Compared to non-meditators, the average sleep duration for a set of Indian meditators was significantly shorter than for non-meditators (5.2 vs. 7.8 hr; *p* < 0.001) (Kaul et al., 2010). Despite the reduced sleep duration of these participants, performance on an attentional task called the psychomotor vigilance task (PVT) was equivalent to non-meditators. Another cross-sectional PSG study reported long-term Tibetan and Theravada Buddhist meditators had significantly reduced sleep time (6.2 vs. 6.7 hr, *p* < 0.001) compared to age- and sex-matched non-meditating controls (Ferrarelli et al., 2013). Our study is consistent with the hypothesis that changes in sleep related to advanced meditation, if they are mediated by improvements in sleep, are due to increased sleep quality as opposed to sleep duration, as we also noticed a decrease in sleep duration in the meditation cohort compared to other cohorts.

Possible mechanisms involved in mediating sleep changes through meditation practices may involve respiratory-brain interactions. The respiratory control neuronal groups in the brain stem have widespread connections. There is correlated cortical and limbic neuronal activity during respiration (Herrero et al., 2018). During volitionally paced breathing, intracranial EEG-breath coherence increases in a frontotemporal-insular network, and during attention to breathing, there is increased coherence in the anterior cingulate, premotor, insular, and hippocampal cortices (Herrero et al., 2018). Slow-deep breathing is a common theme in advanced meditation. As noted above, the respiratory signal permeates the brain, including the hippocampus, but one key target may be the locus coeruleus (Melnychuk et al., 2018). This cell group exclusively provides norepinephrine to the cerebral cortex, and activation of this system reliably induces sleep fragmentation and inhibits sleep spindles (Osorio-Forero et al., 2021, 2022). Modulation of locus coeruleus activity by meditation-related breathing patterns (Melnychuk et al., 2018, 2021) could ultimately enhance spindles or slow-wave activity, which are key components of the BAI. Meditation practices also alter the activation of the anterior cingulate and insular cortices (Fox et al., 2016), which are important generators of the slow oscillations underlying deep non-rapid eye movement sleep. Activation of the carotid body results in sympathetic excitation (Da Silva et al., 2019; Kim & Polotsky, 2020; Moraes et al., 2015), which in turn is associated with arousals and sleep fragmentation. Slow breathing practices also reduce the slope of the hypoxic ventilatory response, reflecting carotid body sensitization (Bernardi et al., 2007; Critchley et al., 2015; Spicuzza et al., 2000).

Greater cortical neural synchronization, both in wakefulness and sleep, is hypothesized to play a role in meditators’ increased cognitive and emotional health. However, it is currently unknown whether meditation is the mediating factor for these improvements, as “psychologically healthy” individuals may be more favorably predisposed towards meditating (Delmonte, 1984). In previous studies, brain age algorithms register lower BAI in neurologically healthy people compared to diseased people (Paixao et al., 2020). Other factors (diet, exercise, genetics) might influence the decision to start meditating or otherwise be correlated. We cannot determine the strength of the direct effect of meditation on BA without knowing the relative strength of these covariates.

Regarding emotional well-being, we identified increased positive affective, friendship and emotional support from pre- to post-meditation retreat, as well as a reduction in perceived stress. We propose that these outcomes are attributed to an enhanced sense of unity, fostered by community and spiritual connections that transcend verbal communication. According to prior research on the experience of mystical states, meaning altered states of consciousness characterized by a profound sense of peace, rhythmic chanting induced mystical states in 60% of participants (Perry et al., 2021). We believe that participants in the study experienced mystical states that enhanced experiences of connection to others, despite not speaking with the other participants during the program. Prior research on the participative benefits of mindfulness meditation supports the findings of decreased loneliness (Creswell et al., 2012) and increased self-compassion (Kozasa et al., 2015).

Our results are based on a re-trained brain age model, which only uses the frontal EEG channels due to the limited electrodes of the devices, rather than using frontal + central + occipital channels in the original brain age paper (Sun et al., 2019). The equivalent of the frontal-only model is supported by the fact that sleep is a global brain event; hence, the features from frontal/central/occipital channels are highly correlated and contain similar information about brain aging. In another study using sleep EEG to predict cognition (Adra et al., 2023), the performance of using frontal channels only is similar to using frontal + central + occipital channels. In a follow-up study of sleep EEG–based brain age in exercise (Ouyang et al., 2024), the Dreem device was used, and a frontal-only brain age model was also re-trained.

### Limitations and Future Research

We cannot definitively conclude that the difference in brain age index is causal because we do not have baseline data before starting meditation. Thus, we cannot fully separate the effects of meditation from those of selection bias, lifestyle choices, diet, or other confounding factors. Future studies should attempt to establish whether there exists a cause-and-effect relationship between meditation and reduced BAI. In addition, the lack of a clear longitudinal effect of years of meditation on brain age index suggests that self-selection bias may be contributing to the lower brain age in this cohort of meditators. Thus, it is unknown whether people who choose to practice meditation are predisposed towards having a lower brain age index, which should be studied in the future. Another limitation is that this sample of meditators, who opted to participate in a study addressing the neural effects of a silent meditation retreat, may create a biased sample due to self-selection. Additionally, a large percentage of participants listed English as a second language, possibly affecting scores on pronunciation or spelling batteries. Furthermore, the high level of education among participants introduces an additional confounding factor, as 93% of meditators in this study hold a bachelor’s, master’s, or other advanced degree beyond high school. The results of this study may not be generalizable to cohorts practicing different meditation practices. Changes from the retreat may be too minute to detect with standard cognitive testing because measurable brain changes take time, and this study aimed to detect changes after a short meditation retreat. Lastly, the frontal-only brain age model is not as widely validated as the original brain age model using frontal, central, and occipital channels.

## Supplementary Information

Below is the link to the electronic supplementary material.

Supplementary file1 (DOCX 7564 KB)

## Data Availability

Data and code are available for download at: https://bdsp.io/content/meditate/1.0/

## Abbreviations

BAI: Brain age index
EEG: Electroencephalography
MGH: Massachusetts General Hospital
PSG: Polysomnography
HC: Healthy control
MCI: Mild cognitive impairment

## Declarations

### Use of Artificial Intelligence

No artificial intelligence tools were used for manuscript preparation.

## References

[CR1] Adra, N., Dümmer, L. W., Paixao, L., Tesh, R. A., Sun, H., Ganglberger, W., Westmeijer, M., Da Silva Cardoso, M., Kumar, A., Ye, E., Henry, J., Cash, S. S., Kitchener, E., Leveroni, C. L., Au, R., Rosand, J., Salinas, J., Lam, A. D., Thomas, R. J., & Westover, M. B. (2023). Decoding information about cognitive health from the brainwaves of sleep. *Scientific Reports,**13*(1), 11448. 10.1038/s41598-023-38310-1 37454163 10.1038/s41598-023-37128-7 PMC10349883

[CR2] Afonso, R. F., Kraft, I., Aratanha, M. A., & Kozasa, E. H. (2020). Neural correlates of meditation: A review of structural and functional MRI studies. *Frontiers in Bioscience,**12*(1), 92–115. 10.2741/S542 10.2741/S542 32114450

[CR3] Al Zoubi, O., Ki Wong, C., Kuplicki, R. T., Yeh, H. W., Mayeli, A., Refai, H., Paulus, M., & Bodurka, J. (2018). Predicting age from brain EEG signals-A machine learning approach. *Frontiers in Aging Neuroscience,**10*, 184. 10.3389/fnagi.2018.00184 30013472 10.3389/fnagi.2018.00184 PMC6036180

[CR4] Baecker, L., Garcia-Dias, R., Vieira, S., Scarpazza, C., & Mechelli, A. (2021). Machine learning for brain age prediction: Introduction to methods and clinical applications. *eBioMedicine,**72*, 103600. 10.1016/j.ebiom.2021.103600 34614461 10.1016/j.ebiom.2021.103600 PMC8498228

[CR5] Baghernezhad, S., & Daliri, M. R. (2024). Age-related changes in human brain functional connectivity using graph theory and machine learning techniques in resting-state fMRI data. *GeroScience,**46*(5), 5303–5320. 10.1007/s11357-024-01128-w 38499956 10.1007/s11357-024-01128-w PMC11336041

[CR6] Bernardi, L., Passino, C., Spadacini, G., Bonfichi, M., Arcaini, L., Malcovati, L., Bandinelli, G., Schneider, A., Keyl, C., Feil, P., Greene, R. E., & Bernasconi, C. (2007). Reduced hypoxic ventilatory response with preserved blood oxygenation in yoga trainees and Himalayan Buddhist monks at altitude: Evidence of a different adaptive strategy? *European Journal of Applied Physiology,**99*(5), 511–518. 10.1007/s00421-006-0373-8 17206440 10.1007/s00421-006-0373-8

[CR7] Black, D. S., O’Reilly, G. A., Olmstead, R., Breen, E. C., & Irwin, M. R. (2015). Mindfulness meditation and improvement in sleep quality and daytime impairment among older adults with sleep disturbances: A randomized clinical trial. *JAMA Internal Medicine,**175*(4), 494. 10.1001/jamainternmed.2014.8081 25686304 10.1001/jamainternmed.2014.8081 PMC4407465

[CR8] Britton, W. B., Lindahl, J. R., Cahn, B. R., Davis, J. H., & Goldman, R. E. (2014). Awakening is not a metaphor: The effects of Buddhist meditation practices on basic wakefulness. *Annals of the New York Academy of Sciences,**1307*(1), 64–81. 10.1111/nyas.12279 24372471 10.1111/nyas.12279 PMC4054695

[CR9] Carrier, J., Land, S., Buysse, D. J., Kupfer, D. J., & Monk, T. H. (2001). The effects of age and gender on sleep EEG power spectral density in the middle years of life (ages 20–60 years old). *Psychophysiology,**38*(2), 232–242. 10.1111/1469-8986.3820232 11347869

[CR10] Chandran, V., Bermúdez, M. L., Koka, M., Chandran, B., Pawale, D., Vishnubhotla, R., Alankar, S., Maturi, R., Subramaniam, B., & Sadhasivam, S. (2021). Large-scale genomic study reveals robust activation of the immune system following advanced Inner Engineering meditation retreat. *Proceedings of the National Academy of Sciences of the United States of America,**118*(51), e2110455118. 10.1073/pnas.2110455118 34907015 10.1073/pnas.2110455118 PMC8713789

[CR11] Creswell, J. D., Irwin, M. R., Burklund, L. J., Lieberman, M. D., Arevalo, J. M., Ma, J., Breen, E. C., & Cole, S. W. (2012). Mindfulness-based stress reduction training reduces loneliness and pro-inflammatory gene expression in older adults: A small randomized controlled trial. *Brain Behavior and Immunity,**26*(7), 1095–1101. 10.1016/j.bbi.2012.07.006 22820409 10.1016/j.bbi.2012.07.006 PMC3635809

[CR12] Critchley, H. D., Nicotra, A., Chiesa, P. A., Nagai, Y., Gray, M. A., Minati, L., & Bernardi, L. (2015). Slow breathing and hypoxic challenge: Cardiorespiratory consequences and their central neural substrates. *PLoS ONE,**10*(5), e0127082. 10.1371/journal.pone.0127082 25973923 10.1371/journal.pone.0127082 PMC4431729

[CR13] Delmonte, M. M. (1984). Electrocortical activity and related phenomena associated with meditation practice: A literature review. *International Journal of Neuroscience,**24*(3–4), 217–231. 10.3109/00207458409089810 6392127 10.3109/00207458409089810

[CR14] De Melo Reis, R. A., Isaac, A. R., Freitas, H. R., de Almeida, M. M., Schuck, P. F., Ferreira, G. C., Andrade-da-Costa, B. L. D. S., & Trevenzoli, I. H. (2021). Quality of life and a surveillant endocannabinoid system. *Frontiers in Neuroscience,**15*, 747229. 10.3389/fnins.2021.747229 34776851 10.3389/fnins.2021.747229 PMC8581450

[CR15] Farras-Permanyer, L., Mancho-Fora, N., Montalà-Flaquer, M., Bartrés-Faz, D., Vaqué-Alcázar, L., Peró-Cebollero, M., & Guàrdia-Olmos, J. (2019). Age-related changes in resting-state functional connectivity in older adults. *Neural Regeneration Research,**14*(9), 1544–1555. 10.4103/1673-5374.255976 31089053 10.4103/1673-5374.255976 PMC6557095

[CR16] Ferrarelli, F., Smith, R., Dentico, D., Riedner, B. A., Zennig, C., Benca, R. M., Lutz, A., Davidson, R. J., & Tononi, G. (2013). Experienced mindfulness meditators exhibit higher parietal-occipital EEG gamma activity during NREM sleep. *PLoS ONE,**8*(8), e73417. 10.1371/journal.pone.0073417 24015304 10.1371/journal.pone.0073417 PMC3756031

[CR17] Fox, K. C., Dixon, M. L., Nijeboer, S., Girn, M., Floman, J. L., Lifshitz, M., Ellamil, M., Sedlmeier, P., & Christoff, K. (2016). Functional neuroanatomy of meditation: A review and meta-analysis of 78 functional neuroimaging investigations. *Neuroscience and Biobehavioral Reviews,**65*, 208–228. 10.1016/j.neubiorev.2016.03.021 27032724 10.1016/j.neubiorev.2016.03.021

[CR18] Fox, K. C., Nijeboer, S., Dixon, M. L., Floman, J. L., Ellamil, M., Rumak, S. P., Sedlmeier, P., & Christoff, K. (2014). Is meditation associated with altered brain structure? A systematic review and meta-analysis of morphometric neuroimaging in meditation practitioners. *Neuroscience and Biobehavioral Reviews,**43*, 48–73. 10.1016/j.neubiorev.2014.03.016 24705269 10.1016/j.neubiorev.2014.03.016

[CR19] Fox, K. C. R., & Cahn, B. R. (2020). Meditation and the brain in health and disease. In M. Farias, D. Brazier, & M. Lalljee (Eds.),* Oxford University Press eBooks*. 10.1093/oxfordhb/9780198808640.013.23

[CR20] Fox, R. S., Zhang, M., Amagai, S., Bassard, A., Dworak, E. M., Han, Y. C., Kassanits, J., Miller, C. H., Nowinski, C. J., Giella, A. K., Stoeger, J. N., Swantek, K., Hook, J. N., & Gershon, R. C. (2022). Uses of the NIH Toolbox® in clinical samples: A scoping review. *Neurology: Clinical Practice, 12*(4), 307–319. 10.1212/CPJ.0000000000200060 10.1212/CPJ.0000000000200060 PMC9647815 36382124

[CR21] Gál, É., Ștefan, S., & Cristea, I. A. (2021). The efficacy of mindfulness meditation apps in enhancing users’ well-being and mental health related outcomes: A meta-analysis of randomized controlled trials. *Journal of Affective Disorders,**279*, 131–142. 10.1016/j.jad.2020.09.134 33049431 10.1016/j.jad.2020.09.134

[CR22] Gao, J., Fan, J., Wu, B. W., Halkias, G. T., Chau, M., Fung, P. C., Chang, C., Zhang, Z., Hung, Y. S., & Sik, H. (2017). Repetitive religious chanting modulates the late-stage brain response to fear- and stress-provoking pictures. *Frontiers in Psychology,**7*, 2055. 10.3389/fpsyg.2016.02055 28119651 10.3389/fpsyg.2016.02055 PMC5223166

[CR23] Gershon, R. C., Wagster, M. V., Hendrie, H. C., Fox, N. A., Cook, K. F., & Nowinski, C. J. (2013). NIH toolbox for assessment of neurological and behavioral function. *Neurology,**80*(11 Supplement 3), S2–S6. 10.1212/WNL.0b013e3182872e5f 23479538 10.1212/WNL.0b013e3182872e5f PMC3662335

[CR24] Gomutbutra, P., Yingchankul, N., Chattipakorn, N., Chattipakorn, S., & Srisurapanont, M. (2020). The effect of mindfulness-based intervention on brain-derived neurotrophic factor (BDNF): A systematic review and meta-analysis of controlled trials. *Frontiers in Psychology,**11*, 2209. 10.3389/fpsyg.2020.02209 33041891 10.3389/fpsyg.2020.02209 PMC7522212

[CR25] Gong, H., Ni, C. X., Liu, Y. Z., Zhang, Y., Su, W. J., Lian, Y. J., Peng, W., & Jiang, C. L. (2016). Mindfulness meditation for insomnia: A meta-analysis of randomized controlled trials. *Journal of Psychosomatic Research,**89*, 1–6. 10.1016/j.jpsychores.2016.07.016 27663102 10.1016/j.jpsychores.2016.07.016

[CR26] Grandchamp, R., & Delorme, A. (2011). Single-trial normalization for event-related spectral decomposition reduces sensitivity to noisy trials. *Frontiers in Psychology,**2*, 236. 10.3389/fpsyg.2011.00236 21994498 10.3389/fpsyg.2011.00236 PMC3183439

[CR27] Hadar, P. N., Westmeijer, M., Sun, H., Meulenbrugge, E. J., Jing, J., Paixao, L., Tesh, R., Da Silva Cardoso, M., Arnal, P., Au, R., Shin, C., Kim, S., Thomas, R. J., Cash, S. S., & Westover, M. B. (2024). Epilepsy is associated with the accelerated aging of brain activity in sleep. *Frontiers in Physiology,**15*, 1458592. 39668843 10.3389/fphys.2024.1458592 PMC11634596

[CR28] Herrero, J. L., Khuvis, S., Yeagle, E., Cerf, M., & Mehta, A. D. (2018). Breathing above the brain stem: Volitional control and attentional modulation in humans. *Journal of Neurophysiology,**119*(1), 145–159. 10.1152/jn.00551.2017 28954895 10.1152/jn.00551.2017 PMC5866472

[CR29] Ho, D. E., Imai, K., King, G., & Stuart, E. A. (2007). Matching as nonparametric preprocessing for reducing model dependence in parametric causal inference. *Political Analysis,**15*(3), 199–236. 10.1093/pan/mpl013

[CR30] Hodes, R. J., Insel, T. R., & Landis, S. C. (2013). The NIH Toolbox: Setting a standard for biomedical research. *Neurology*, *80*(11_supplement_3). 10.1212/wnl.0b013e3182872e90 10.1212/WNL.0b013e3182872e90 PMC3662338 23479536

[CR31] Hogan, J., Sun, H., Paixao, L., Westmeijer, M., Sikka, P., Jin, J., Tesh, R., Cardoso, M., Cash, S. S., Akeju, O., Thomas, R., & Westover, M. B. (2021). Night-to-night variability of sleep electroencephalography-based brain age measurements. *Clinical Neurophysiology,**132*(1), 1–12. 10.1016/j.clinph.2020.09.029 33248430 10.1016/j.clinph.2020.09.029 PMC7855943

[CR32] Kanchibhotla, D., Parekh, S. G., Harsora, P., & Kulkarni, S. (2021). Improvements in sleep quality and duration following a meditation retreat: An open-trial pilot study. *Sleep and Vigilance,**5*(2), 275–280. 10.1007/s41782-021-00162-4

[CR33] Kaul, P., Passafiume, J., Sargent, R. C., & O’Hara, B. F. (2010). Meditation acutely improves psychomotor vigilance, and may decrease sleep need. *Behavioral and Brain Functions,**6*, 47. 10.1186/1744-9081-6-47 20670413 10.1186/1744-9081-6-47 PMC2919439

[CR34] Kaur, C., & Singh, P. (2015). EEG derived neuronal dynamics during meditation: Progress and challenges. *Advances in Preventive Medicine,**2015*(1), 614723. 10.1155/2015/614723 26770834 10.1155/2015/614723 PMC4684838

[CR35] Khoury, B., Knäuper, B., Schlosser, M., Carrière, K., & Chiesa, A. (2017). Effectiveness of traditional meditation retreats: A systematic review and meta-analysis. *Journal of Psychosomatic Research,**92*, 16–25. 10.1016/j.jpsychores.2016.11.006 27998508 10.1016/j.jpsychores.2016.11.006

[CR36] Kim, L. J., & Polotsky, V. Y. (2020). Carotid body and metabolic syndrome: Mechanisms and potential therapeutic targets. *International Journal of Molecular Sciences,**21*(14), 5117. 10.3390/ijms21145117 32698380 10.3390/ijms21145117 PMC7404212

[CR37] Kozasa, E. H., Lacerda, S. S., Menezes, C., Wallace, B. A., Radvany, J., Mello, L. E. a. M., & Sato, J. R. (2015). Effects of a 9-day Shamatha Buddhist meditation retreat on attention, mindfulness and self-compassion in participants with a broad range of meditation experience. *Mindfulness*, *6*(6), 1235–1241. 10.1007/s12671-015-0385-8

[CR38] Kurth, F., Cherbuin, N., & Luders, E. (2017). Promising links between meditation and reduced (brain) aging: An attempt to bridge some gaps between the alleged fountain of youth and the youth of the field. *Frontiers in Psychology,**8*, 860. 10.3389/fpsyg.2017.00860 28611710 10.3389/fpsyg.2017.00860 PMC5447722

[CR39] Kurth, F., Strohmaier, S., & Luders, E. (2023). Reduced age-related gray matter loss in the orbitofrontal cortex in long-term meditators. *Brain Sciences,**13*(12), 1677. 10.3390/brainsci13121677 38137125 10.3390/brainsci13121677 PMC10741700

[CR40] Laneri, D., Schuster, V., Dietsche, B., Jansen, A., Ott, U., & Sommer, J. (2016). Effects of long-term mindfulness meditation on brain’s white matter microstructure and its aging. *Frontiers in Aging Neuroscience,**7*, 254. 10.3389/fnagi.2015.00254 26834624 10.3389/fnagi.2015.00254 PMC4712309

[CR41] Larsen, L. H., Moe, K. E., Vitiello, M. V., & Prinz, P. N. (1995). Age trends in the sleep EEG of healthy older men and women. *Journal of Sleep Research,**4*(3), 160–172. 10.1111/j.1365-2869.1995.tb00164.x 10607155 10.1111/j.1365-2869.1995.tb00165.x

[CR42] Lee, K. C. G., Gao, J., Leung, H. K., Wu, B. W. Y., Roberts, A., Thach, T. Q., & Sik, H. H. (2024). Modulating consciousness through awareness training program and its impacts on psychological stress and age-related gamma waves. *Brain Sciences,**14*(1), 91. 10.3390/brainsci14010091 38248306 10.3390/brainsci14010091 PMC10813729

[CR43] Leone, M. J., Sun, H., Boutros, C. L., Liu, L., Ye, E., Sullivan, L., Thomas, R. J., Robbins, G. K., Mukerji, S. S., & Westover, M. B. (2021). HIV increases sleep-based brain age despite antiretroviral therapy. *Sleep,**44*(8), zsab058. 10.1093/sleep/zsab058 33783511 10.1093/sleep/zsab058 PMC8361332

[CR44] Luders, E., Cherbuin, N., & Gaser, C. (2016). Estimating brain age using high-resolution pattern recognition: Younger brains in long-term meditation practitioners. *NeuroImage,**134*, 508–513. 10.1016/j.neuroimage.2016.04.007 27079530 10.1016/j.neuroimage.2016.04.007

[CR45] Lund, M. J., Alnæs, D., de Lange, A. G., Andreassen, O. A., Westlye, L. T., & Kaufmann, T. (2022). Brain age prediction using fMRI network coupling in youths and associations with psychiatric symptoms. *NeuroImage: Clinical,**33*, 102921. 10.1016/j.nicl.2021.102921 10.1016/j.nicl.2021.102921 PMC8718718 34959052

[CR46] Magalhaes, A. A., Oliveira, L., Pereira, M. G., & Menezes, C. B. (2018). Does meditation alter brain responses to negative stimuli? A systematic review. *Frontiers in Human Neuroscience,**12*, 448. 10.3389/fnhum.2018.00448 30483083 10.3389/fnhum.2018.00448 PMC6243128

[CR47] Mander, B. A., Winer, J. R., & Walker, M. P. (2017). Sleep and human aging. *Neuron,**94*(1), 19–36. 10.1016/j.neuron.2017.02.004 28384471 10.1016/j.neuron.2017.02.004 PMC5810920

[CR48] Martires, J., & Zeidler, M. (2015). The value of mindfulness meditation in the treatment of insomnia. *Current Opinion in Pulmonary Medicine,**21*(6), 547–552. 10.1097/MCP.0000000000000207 26390335 10.1097/MCP.0000000000000207

[CR49] Maruthai, N., Nagendra, R. P., Sasidharan, A., Srikumar, S., Datta, K., Uchida, S., & Kutty, B. M. (2016). Senior Vipassana meditation practitioners exhibit distinct REM sleep organization from that of novice meditators and healthy controls. *International Review of Psychiatry,**28*(3), 279–287. 10.3109/09540261.2016.1159949 27055575 10.3109/09540261.2016.1159949

[CR50] Mason, L. I., Alexander, C. N., Travis, F. T., Marsh, G., Orme-Johnson, D. W., Gackenbach, J., Mason, D. C., Rainforth, M., & Walton, K. G. (1997). Electrophysiological correlates of higher states of consciousness during sleep in long-term practitioners of the Transcendental meditation program. *Sleep,**20*(2), 102–110. 10.1093/sleep/20.2.102 9143069 10.1093/sleep/20.2.102

[CR51] Melnychuk, M. C., Dockree, P. M., O’Connell, R. G., Murphy, P. R., Balsters, J. H., & Robertson, I. H. (2018). Coupling of respiration and attention via the locus coeruleus: Effects of meditation and pranayama. *Psychophysiology*, *55*(9). 10.1111/psyp.13091 10.1111/psyp.13091 29682753

[CR52] Melnychuk, M. C., Robertson, I. H., Plini, E. R. G., & Dockree, P. M. (2021). A bridge between the breath and the brain: Synchronization of respiration, a pupillometric marker of the locus coeruleus, and an EEG marker of attentional control state. *Brain Sciences,**11*(10), 1324. 10.3390/brainsci11101324 34679389 10.3390/brainsci11101324 PMC8534189

[CR53] Millar, P. R., Luckett, P. H., Gordon, B. A., Benzinger, T. L. S., Schindler, S. E., Fagan, A. M., Cruchaga, C., Bateman, R. J., Allegri, R., Jucker, M., Lee, J. H., Mori, H., Salloway, S. P., Yakushev, I., Morris, J. C., Ances, B. M., & Network, D. I. A. (2022). Predicting brain age from functional connectivity in symptomatic and preclinical Alzheimer disease. *NeuroImage,**256*, 119228. 10.1016/j.neuroimage.2022.119228 35452806 10.1016/j.neuroimage.2022.119228 PMC9178744

[CR54] Moraes, D. J., Machado, B. H., & Paton, J. F. (2015). Carotid body overactivity induces respiratory neurone channelopathy contributing to neurogenic hypertension. *The Journal of Physiology,**593*(14), 3055–3063. 10.1113/JP270423 25900825 10.1113/JP270423 PMC4532526

[CR55] Nagendra, R. P., Maruthai, N., & Kutty, B. M. (2012). Meditation and its regulatory role on sleep. *Frontiers in Neurology,**3*, 54. 10.3389/fneur.2012.00054 22529834 10.3389/fneur.2012.00054 PMC3328970

[CR56] Nanthakwang, N., Siviroj, P., Matanasarawoot, A., Sapbamrer, R., Lerttrakarnnon, P., & Awiphan, R. (2020). Effectiveness of deep breathing and body scan meditation combined with music to improve sleep quality and quality of life in older adults. *The Open Public Health Journal,**13*(1), 232–239. 10.2174/1874944502013010232

[CR57] Neuendorf, R., Wahbeh, H., Chamine, I., Yu, J., Hutchison, K., & Oken, B. S. (2015). The effects of mind-body interventions on sleep quality: A systematic review. *Evidence-Based Complementary and Alternative Medicine,**2015*, 902708. 10.1155/2015/902708 26161128 10.1155/2015/902708 PMC4487927

[CR58] Ng, T. K. S., Fam, J., Feng, L., Cheah, I. K., Tan, C. T., Nur, F., Wee, S. T., Goh, L. G., Chow, W. L., Ho, R. C., Kua, E. H., Larbi, A., & Mahendran, R. (2020). Mindfulness improves inflammatory biomarker levels in older adults with mild cognitive impairment: A randomized controlled trial. *Translational Psychiatry,**10*(1), 21. 10.1038/s41398-020-0696-y 32066726 10.1038/s41398-020-0696-y PMC7026149

[CR59] Ong, J. C., Manber, R., Segal, Z., Xia, Y., Shapiro, S., & Wyatt, J. K. (2014). A randomized controlled trial of mindfulness meditation for chronic insomnia. *Sleep,**37*(9), 1553–1563. 10.5665/sleep.4010 25142566 10.5665/sleep.4010 PMC4153063

[CR60] Osorio-Forero, A., Cardis, R., Vantomme, G., Guillaume-Gentil, A., Katsioudi, G., Devenoges, C., Fernandez, L. M. J., & Lüthi, A. (2021). Noradrenergic circuit control of non-REM sleep substates. *Current Biology,**31*(22), 5009-5023.e7. 10.1016/j.cub.2021.09.041 34648731 10.1016/j.cub.2021.09.041

[CR61] Osorio-Forero, A., Cherrad, N., Banterle, L., Fernandez, L. M. J., & Lüthi, A. (2022). When the locus coeruleus speaks up in sleep: Recent insights, emerging perspectives. *International Journal of Molecular Sciences,**23*(9), 5028. 10.3390/ijms23095028 35563419 10.3390/ijms23095028 PMC9099715

[CR62] Ouyang, A., Zhang, C., Adra, N., Tesh, R. A., Sun, H., Lei, D., Jing, J., Fan, P., Paixao, L., Ganglberger, W., Briggs, L., Salinas, J., Bevers, M. B., Wrann, C. D., Chemali, Z., Fricchione, G., Thomas, R. J., Rosand, J., Tanzi, R. E., & Westover, M. B. (2024). Effects of aerobic exercise on brain age and health in middle-aged and older adults: A single-arm pilot clinical trial. *Life,**14*(7), 855. 10.3390/life14070855 39063609 10.3390/life14070855 PMC11278044

[CR63] Paixao, L., Sikka, P., Sun, H., Jain, A., Hogan, J., Thomas, R., & Westover, M. B. (2020). Excess brain age in the sleep electroencephalogram predicts reduced life expectancy. *Neurobiology of Aging,**88*, 150–155. 10.1016/j.neurobiolaging.2019.12.015 31932049 10.1016/j.neurobiolaging.2019.12.015 PMC7085452

[CR64] Perez-Diaz, O., Góngora, D., González-Mora, J. L., Rubia, K., Barrós-Loscertales, A., & Hernández, S. E. (2024). Enhanced amygdala-anterior cingulate white matter structural connectivity in Sahaja Yoga Meditators. *PLoS ONE,**19*(3), e0301283. 10.1371/journal.pone.0301283 38547155 10.1371/journal.pone.0301283 PMC10977753

[CR65] Pernet, C. R., Belov, N., Delorme, A., & Zammit, A. (2021). Mindfulness related changes in grey matter: A systematic review and meta-analysis. *Brain Imaging and Behavior,**15*(5), 2720–2730. 10.1007/s11682-021-00453-4 33624219 10.1007/s11682-021-00453-4 PMC8500886

[CR66] Perry, G., Polito, V., & Thompson, W. F. (2021). Rhythmic chanting and mystical states across traditions. *Brain Sciences,**11*(1), 101. 10.3390/brainsci11010101 33451163 10.3390/brainsci11010101 PMC7828722

[CR67] Prerau, M. J., Brown, R. E., Bianchi, M. T., Ellenbogen, J. M., & Purdon, P. L. (2017). Sleep neurophysiological dynamics through the lens of multitaper spectral analysis. *Physiology,**32*(1), 60–92. 10.1152/physiol.00035.2016 27927806 10.1152/physiol.00062.2015 PMC5343535

[CR68] Purcell, S. M., Manoach, D. S., Demanuele, C., Cade, B. E., Mariani, S., Cox, R., ... & Stickgold, R. (2017). Characterizing sleep spindles in 11,630 individuals from the National Sleep Research Resource. *Nature Communications*, *8*(1), 15930. 10.1038/ncomms15930 10.1038/ncomms15930 PMC5490197 28649997

[CR69] Raman, M., Vishnubhotla, R., Ramay, H. R., Gonçalves, M. C. B., Shin, A. S., Pawale, D., Subramaniam, B., & Sadhasivam, S. (2023). Isha yoga practices, vegan diet, and participation in Samyama meditation retreat: Impact on the gut microbiome & metabolome - a non-randomized trial. *BMC Complementary Medicine and Therapies,**23*(1), 107. 10.1186/s12906-023-03935-8 37020274 10.1186/s12906-023-03935-8 PMC10074366

[CR70] Rao, K. S., Chakraharti, S. K., Dongare, V. S., Chetana, K., Ramirez, C. M., Koka, P. S., & Deb, K. D. (2015). Antiaging effects of an intensive mind and body therapeutic program through enhancement of telomerase activity and adult stem cell counts. *Journal of Stem Cells,**10*(2), 107–125. 27125139

[CR71] Rathore, M., Verma, M., Nirwan, M., Trivedi, S., & Pai, V. (2022). Functional connectivity of prefrontal cortex in various meditation techniques - A mini-review. *International Journal of Yoga,**15*(3), 187–194. 10.4103/ijoy.ijoy_88_22 36949839 10.4103/ijoy.ijoy_88_22 PMC10026337

[CR72] Rodriguez-Larios, J., de Oca, E. A. B. M., & Alaerts, K. (2021). The EEG spectral properties of meditation and mind wandering differ between experienced meditators and novices. *NeuroImage,**245*, 118669. 10.1016/j.neuroimage.2021.118669 34688899 10.1016/j.neuroimage.2021.118669

[CR73] Rusch, H. L., Rosario, M., Levison, L. M., Olivera, A., Livingston, W. S., Wu, T., & Gill, J. M. (2019). The effect of mindfulness meditation on sleep quality: A systematic review and meta-analysis of randomized controlled trials. *Annals of the New York Academy of Sciences,**1445*(1), 5–16. 10.1111/nyas.13996 30575050 10.1111/nyas.13996 PMC6557693

[CR74] Sadhasivam, S., Alankar, S., Maturi, R., Vishnubhotla, R. V., Mudigonda, M., Pawale, D., Narayanan, S., Hariri, S., Ram, C., Chang, T., Renschler, J., Eckert, G., & Subramaniam, B. (2020). Inner Engineering practices and advanced 4-day Isha Yoga retreat are associated with cannabimimetic effects with increased endocannabinoids and short-term and sustained improvement in mental health: A prospective observational study of meditators. *Evidence-Based Complementary and Alternative Medicine,**2020*, 8438272. 10.1155/2020/8438272 32595741 10.1155/2020/8438272 PMC7293737

[CR75] Sadhasivam, S., Alankar, S., Maturi, R., Williams, A., Vishnubhotla, R. V., Hariri, S., Mudigonda, M., Pawale, D., Dubbireddi, S., Packiasabapathy, S., Castelluccio, P., Ram, C., Renschler, J., Chang, T., & Subramaniam, B. (2021). Isha Yoga practices and participation in Samyama program are associated with reduced HbA1C and systemic inflammation, improved lipid profile, and short-term and sustained improvement in mental health: A prospective observational study of meditators. *Frontiers in Psychology,**12*, 659667. 10.3389/fpsyg.2021.659667 34093351 10.3389/fpsyg.2021.659667 PMC8170079

[CR76] Schutte, N. S., Malouff, J. M., & Keng, S. L. (2020). Meditation and telomere length: A meta-analysis. *Psychology & Health,**35*(8), 901–915. 10.1080/08870446.2019.1707827 31903785 10.1080/08870446.2019.1707827

[CR77] Scott, S. B., Graham-Engeland, J. E., Engeland, C. G., Smyth, J. M., Almeida, D. M., Katz, M. J., Lipton, R. B., Mogle, J. A., Munoz, E., Ram, N., & Sliwinski, M. J. (2015). The Effects of Stress on Cognitive Aging, Physiology and Emotion (ESCAPE) project. *BMC Psychiatry,**15*, 146. 10.1186/s12888-015-0497-7 26138700 10.1186/s12888-015-0497-7 PMC4490700

[CR78] Scullin, M. K. (2017). Do older adults need sleep? A review of neuroimaging, sleep, and aging studies. *Current Sleep Medicine Reports,**3*, 204–214. 10.1007/s40675-017-0086-z 29226069 10.1007/s40675-017-0086-z PMC5720383

[CR79] Sone, D., & Beheshti, I. (2022). Neuroimaging-based brain age estimation: A promising personalized biomarker in neuropsychiatry. *Journal of Personalized Medicine,**12*(11), 1850. 10.3390/jpm12111850 36579560 10.3390/jpm12111850 PMC9695293

[CR80] Spicuzza, L., Gabutti, A., Porta, C., Montano, N., & Bernardi, L. (2000). Yoga and chemoreflex response to hypoxia and hypercapnia. *Lancet,**356*(9240), 1495–1496. 10.1016/S0140-6736(00)02881-6 11081541 10.1016/S0140-6736(00)02881-6

[CR81] Stuart, E. A. (2010). Matching methods for causal inference: A review and a look forward. *Statistical Science,**25*(1), 1–21. 10.1214/09-STS313 20871802 10.1214/09-STS313 PMC2943670

[CR82] Sulekha, S., Thennarasu, K., Vedamurthachar, A., Raju, T. R., & Kutty, B. M. (2006). Evaluation of sleep architecture in practitioners of Sudarshan Kriya yoga and Vipassana meditation. *Sleep and Biological Rhythms,**4*(3), 207–214. 10.1111/j.1479-8425.2006.00233.x

[CR83] Sun, H., Paixao, L., Oliva, J. T., Goparaju, B., Carvalho, D. Z., van Leeuwen, K. G., Akeju, O., Thomas, R. J., Cash, S. S., Bianchi, M. T., & Westover, M. B. (2019). Brain age from the electroencephalogram of sleep. *Neurobiology of Aging,**74*, 112–120. 10.1016/j.neurobiolaging.2018.10.016 30448611 10.1016/j.neurobiolaging.2018.10.016 PMC6478501

[CR84] Sun, H., Adra, N., Ayub, M. A., Ganglberger, W., Ye, E., Fernandes, M., Paixao, L., Fan, Z., Gupta, A., Ghanta, M., Moura Junior, V. F., Rosand, J., Westover, M. B., & Thomas, R. J. (2024). Assessing risk of health outcomes from brain activity in sleep: A retrospective cohort study. *Neurology. Clinical Practice,**14*(1), e200225. 10.1212/CPJ.0000000000200225 38173542 10.1212/CPJ.0000000000200225 PMC10759032

[CR85] Takahashi, T., Murata, T., Hamada, T., Omori, M., Kosaka, H., Kikuchi, M., Yoshida, H., & Wada, Y. (2005). Changes in EEG and autonomic nervous activity during meditation and their association with personality traits. *International Journal of Psychophysiology,**55*(2), 199–207. 10.1016/j.ijpsycho.2004.07.004 15649551 10.1016/j.ijpsycho.2004.07.004

[CR86] Thoemmes, F. J., & Kim, E. S. (2011). A systematic review of propensity score methods in the social sciences. *Multivariate Behavioral Research,**46*(1), 90–118. 10.1080/00273171.2011.540475 26771582 10.1080/00273171.2011.540475

[CR87] Thomson, D. J. (1982). Spectrum estimation and harmonic analysis. *Proceedings of the IEEE,**70*, 1055–1096. 10.1109/PROC.1982.12433

[CR88] Van Lutterveld, R., Van Dellen, E., Pal, P., Yang, H., Stam, C. J., & Brewer, J. (2017). Meditation is associated with increased brain network integration. *NeuroImage,**158*, 18–25. 10.1016/j.neuroimage.2017.06.071 28663069 10.1016/j.neuroimage.2017.06.071 PMC5614811

[CR89] Vishnubhotla, R. V., Radhakrishnan, R., Kveraga, K., Deardorff, R., Ram, C., Pawale, D., Wu, Y. C., Renschler, J., Subramaniam, B., & Sadhasivam, S. (2021). Advanced meditation alters resting-state brain network connectivity correlating with improved mindfulness. *Frontiers in Psychology,**12*, 745344. 10.3389/fpsyg.2021.745344 34867626 10.3389/fpsyg.2021.745344 PMC8636330

[CR90] Vishnubhotla, R. V., Wood, P. L., Verma, A., Cebak, J. E., Hariri, S., Mudigonda, M., Alankar, S., Maturi, R., Orui, H., Subramaniam, B., Palwale, D., Renschler, J., & Sadhasivam, S. (2022). Advanced meditation and vegan diet increased acylglycines and reduced lipids associated with improved health: A prospective longitudinal study. *Journal of Integrative and Complementary Medicine,**28*(8), 674–682. 10.1089/jicm.2022.0480 35532984 10.1089/jicm.2022.0480

[CR91] World Health Organization. (2017). *Global action plan on the public health response to dementia 2017–2025*. World Health Organization. https://apps.who.int/iris/handle/10665/259615. Accessed 3/1/2025.

[CR92] World Health Organization. (2018). *Towards a dementia plan: A WHO guide*. World Health Organization. https://apps.who.int/iris/handle/10665/272642. Accessed 3/1/2025.

[CR93] Ye, E., Sun, H., Leone, M. J., Paixao, L., Thomas, R. J., Lam, A. D., & Westover, M. B. (2020). Association of sleep electroencephalography-based brain age index with dementia. *JAMA Network Open,**3*(9), e2017357. 10.1001/jamanetworkopen.2020.17357 32986106 10.1001/jamanetworkopen.2020.17357 PMC7522697

[CR94] Yook, S., Park, H. R., Joo, E. Y., & Kim, H. (2024). Predicting the impact of CPAP on brain health: A study using the sleep EEG-derived brain age index. *Annals of Clinical and Translational Neurology,**11*(5), 1172–1183. 10.1002/acn3.10379 38396240 10.1002/acn3.52032 PMC11093235

