# Supplementary material for: Sleep-Based Brain Age Is Reduced in Advanced Inner Engineering Meditators
Source: Mindfulness (N Y). 2025 May 16;16(6):1675–92. doi: 10.1007/s12671-025-02583-y (PMC12170783; doi:10.1007/s12671-025-02583-y)
Supplement: Supplementary file 1 — Supplementary file1 (DOCX 7564 KB) [file 12671_2025_2583_MOESM1_ESM.docx]

**SUPPLEMENTARY METHODS, ANALYSIS, AND FIGURES**

**Sleep-Based Brain Age is Reduced in Advanced Meditators**

Jayme C. Banks, BA, Sepideh Hariri, PhD, Kestutis Kveraga, PhD, An Ouyang, Kaileigh Gallagher, BS, Syed A. Quadri, MD, Noor Adra, BA, Ryan A. Tesh, BSc, Preeti Upadhyay Reed, MPH, Pierrick J. Arnal, PhD, Robert J. Thomas, MD, M. Brandon Westover, MD, PhD, Haoqi Sun, PhD, Balachundhar Subramaniam, MD, MPH

**Corresponding Author:** M. Brandon Westover, MD, PhD**,** Department of Neurology**,** Beth Israel Deaconess Medical Center**,** 330 Brookline Avenue, Boston, MA 02215 **(**mwestove@bidmc.harvard.edu**;** 617-724-3703)

**Table of Contents**

[Figure S1 2](#_Toc194762190)

[Figure S2a-f 3](#_Toc194762191)

[Figure S3 9](#_Toc194762192)

[Table S1. Unadjusted brain age index (BAI) by group 10](#_Toc194762193)

[Table S2. Time comparison of NIH toolbox scores for the meditation cohort 11](#_Toc194762194)

[Supplemental Analysis 1. Analysis of NIH toolbox (time comparison) using JASP software 12](#_Toc194762195)

[Supplemental Method. Using linear discriminant analysis (LDA) to remove artifacts 12](#_Toc194762196)

[Supplemental Method. Definitions of dementia, MCI, symptomatic, no dementia, and healthy groups from the sleep lab 13](#_Toc194762197)

# Figure S1


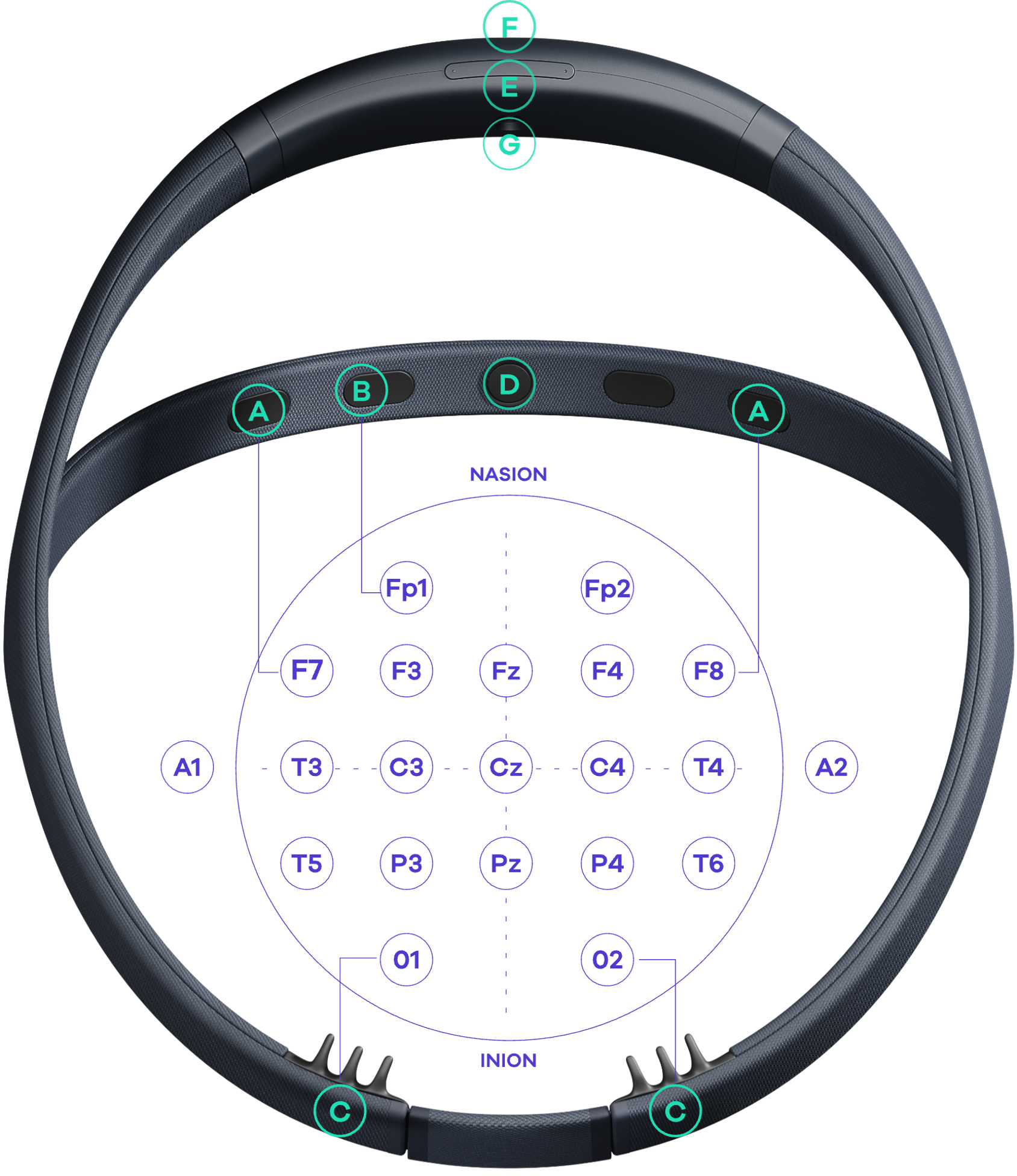


An example Dreem headband, and the electrode locations. The picture is from the Dreem website (now acquired by Beacon): https://beacon.bio/dreem-headband/.

# Figure S2a-f


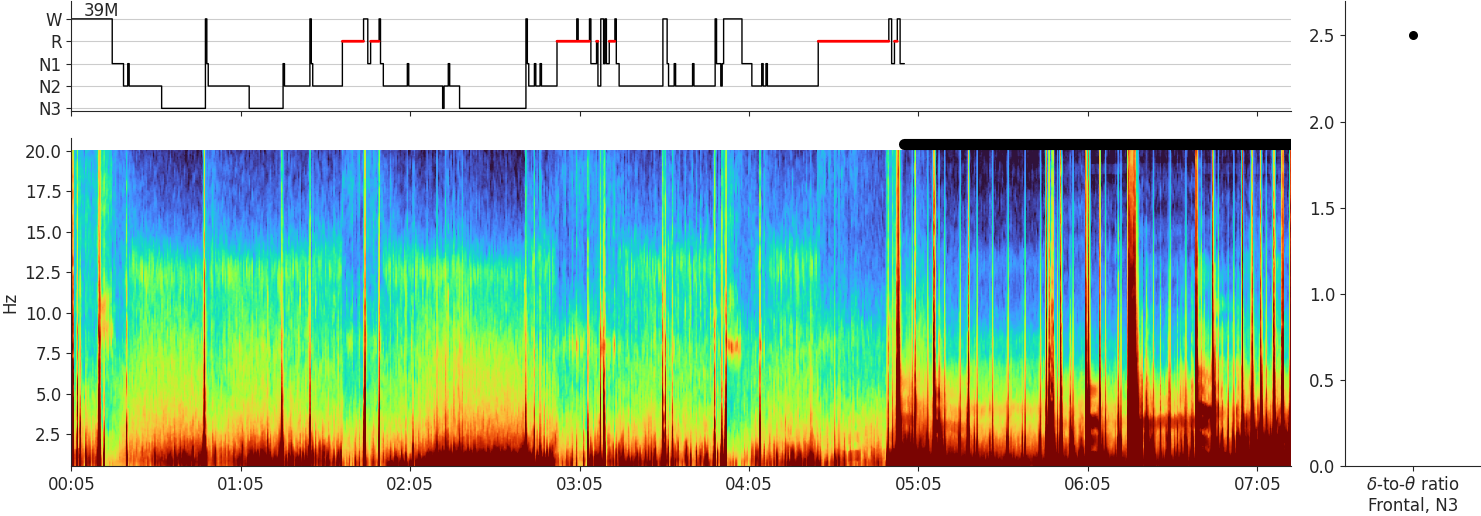


**Figure S2a.** An example spectrogram and hypnogram from a member of the meditation cohort. This is from a 39 year old male with brain age 31.4 years and brain age index -7.6 years. Sleep onset is fast. There are four sleep stage cycles, which are intact, clear, and happened within five hours. The bar on top of the spectrogram indicates detected artifact epochs which are not included in the brain age calculation. Visible features in the spectrogram that correlate with negative BAI include strong delta (1-4 Hz) power during N3 and clear spindles around 12.5 Hz.


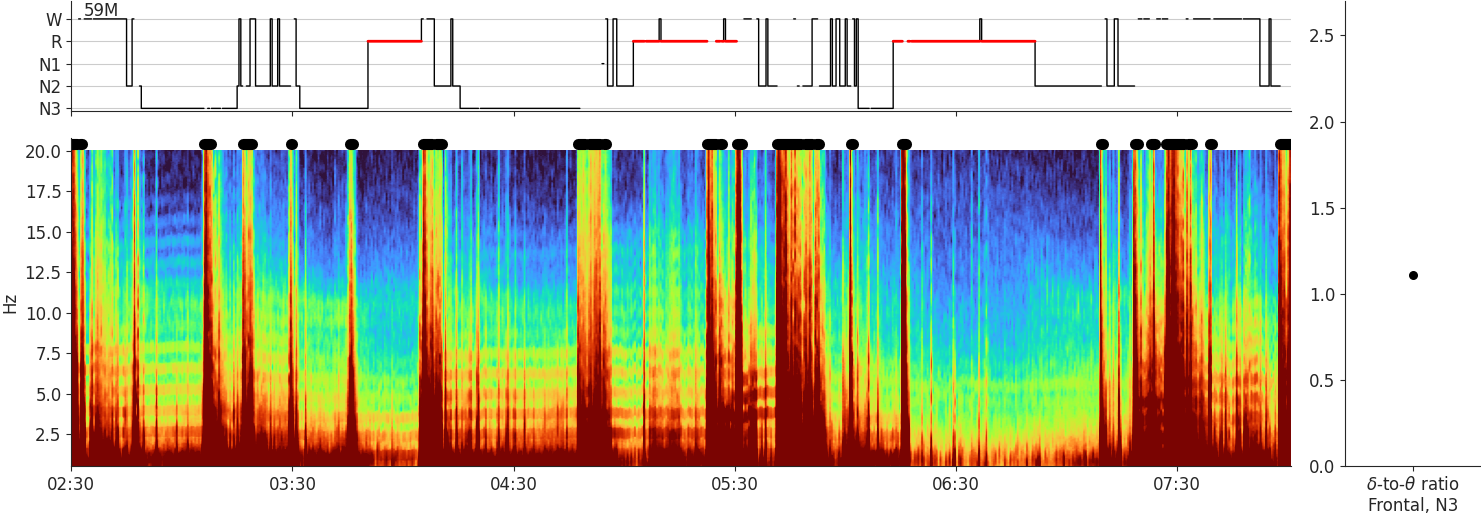


**Figure S2b.** An example spectrogram and hypnogram from a member of the Dreem healthy controls cohort. This is from a 59 year old male with brain age 57.4 years and brain age index -1.6 years. The signal quality is moderate, and several segments are identified as having excessive artifact (black bars). There are also ECG artifacts manifesting as horizontal stripes. Despite the artifacts, one can see the spindle band at around 11 Hz, although broad and vague. Sleep stage cycles are evident despite the frequent artifact.


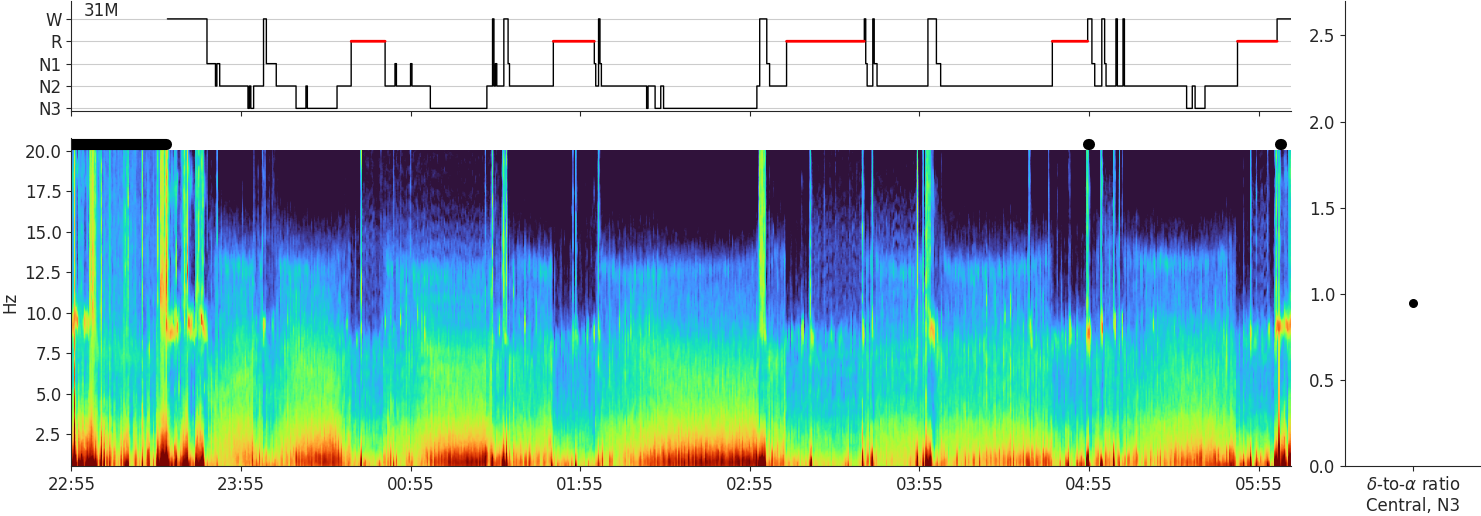


**Figure S2c.** An example sleep EEG spectrogram averaged from the MGH healthy controls cohort. This is from a 31 year old male with brain age 31.8 years and brain age index of +0.8 years. This is an age-appropriate sleep EEG spectrogram for a person in their early 30s, with clear boundaries between sleep stages and rich patterns corresponding to the hypnogram. The alpha peak frequency during the awake (W) stage is at 10 Hz, matching the typical frequency at the third decade.


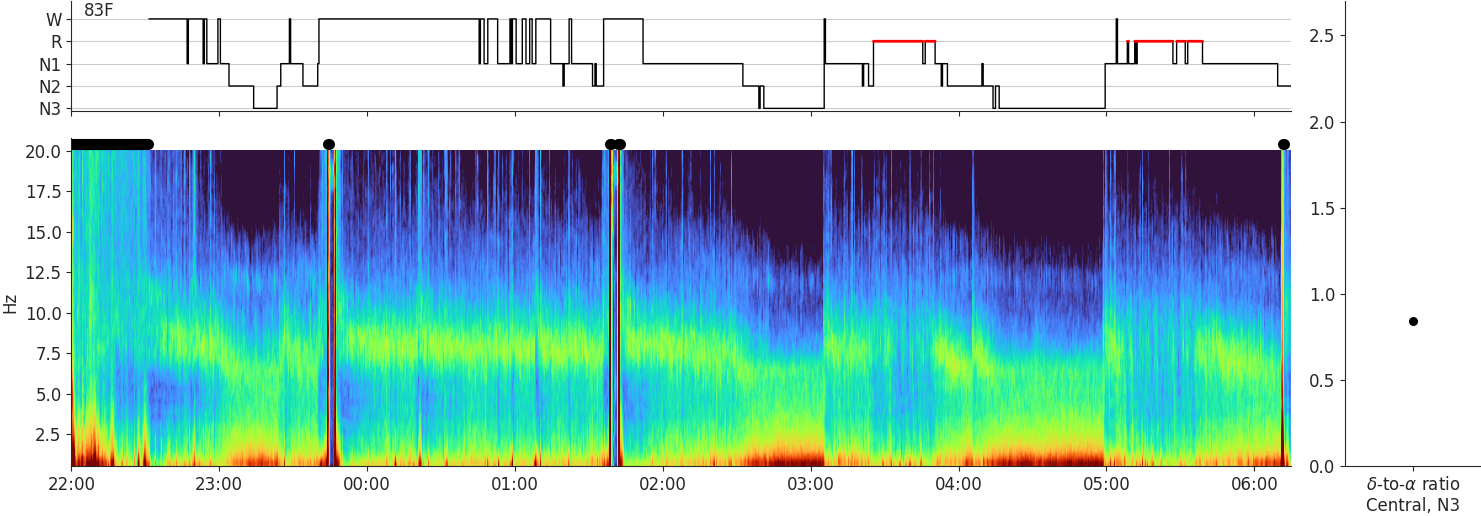


**Figure S2d.** An example sleep EEG spectrogram and hypnogram from a patient in the MGH symptomatic cohort. This is from an 83-year-old female with brain age 85.4 years and brain age index +2.4 years. There is a large amount of wakefulness after sleep onset. The alpha peak frequency during wakefulness (W) is broad and around 8 Hz, at the lower limit of normal. Spindles are not obvious but present. The delta power during N3 is lower than in the spectrogram from the younger patient above.


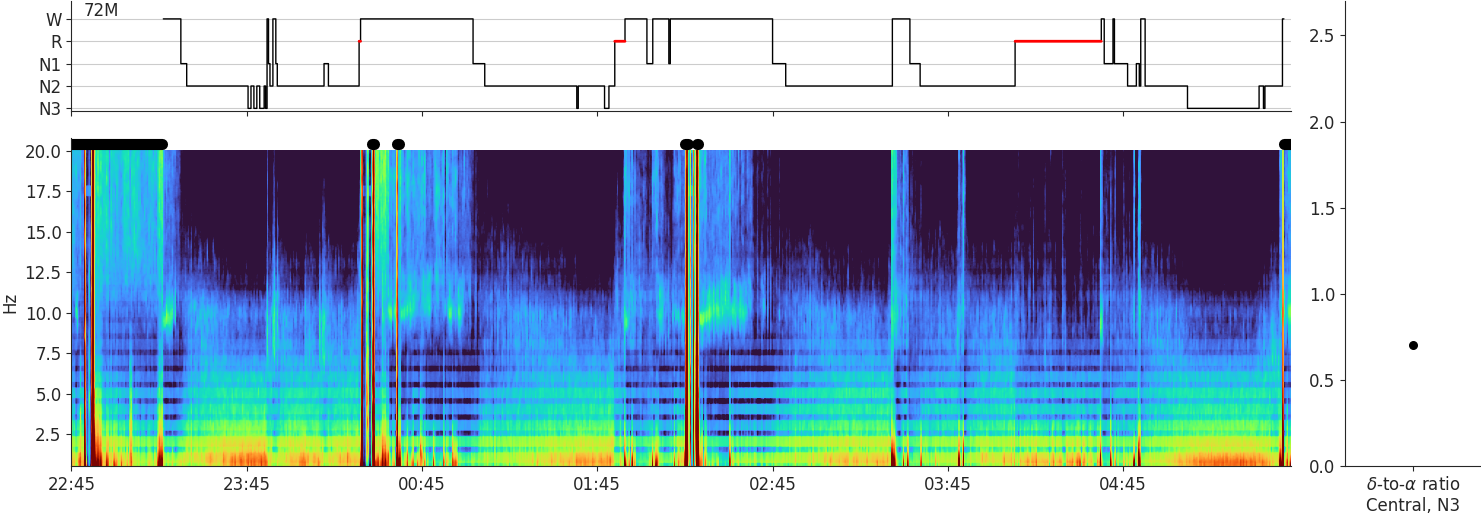


**Figure S2e**. An example sleep EEG spectrogram and its hypnogram from the MGH mild cognitive impairment cohort. This is from a 72 year old male with brain age 76.6 years and brain age index +4.6 years. There is a high amount of wake after sleep onset. There are ECG artifacts represented as horizontal stripes. The overall power (based on the color) is low, especially the delta power.


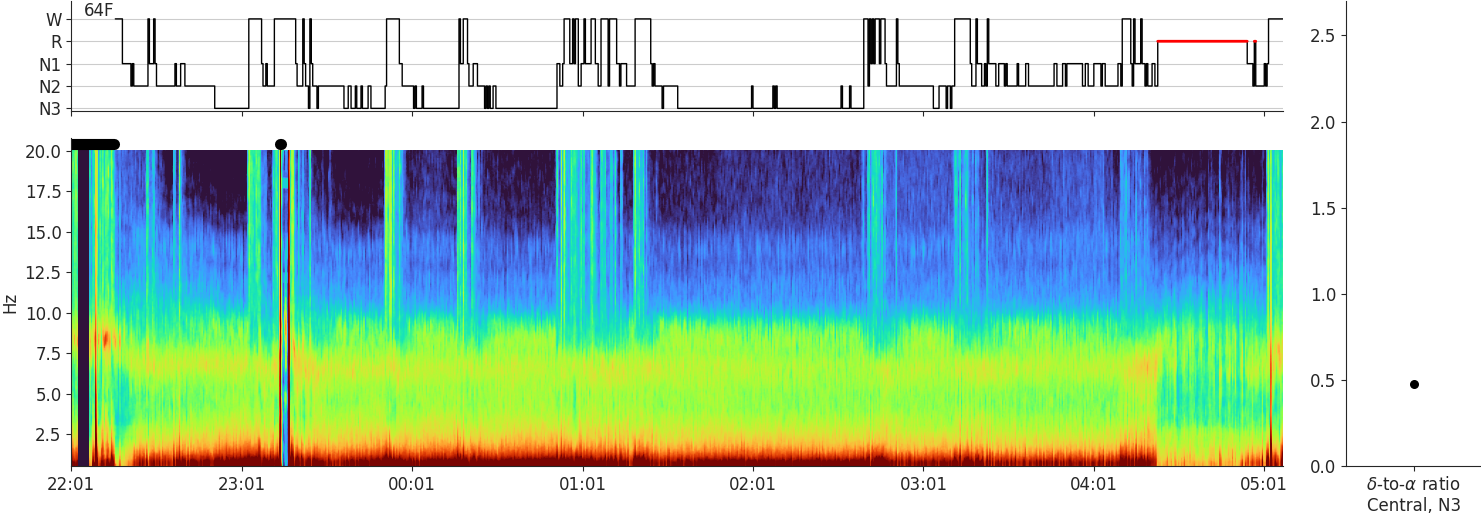


**Figure S2f.** An example sleep EEG spectrogram and hypnogram from the MGH dementia cohort. This is form a 64 year old female with brain age of 72.2 years and brain age index +8.2 years. This is an “old-looking” sleep EEG spectrogram in terms of a relatively invariant pattern presenting for most of the night. The constant activity around 7 to 7.5 Hz is abnormal. The hypnogram is fragmented.

# Figure S3


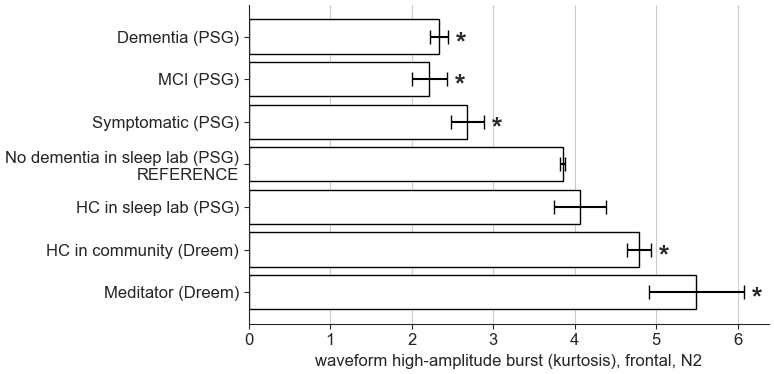

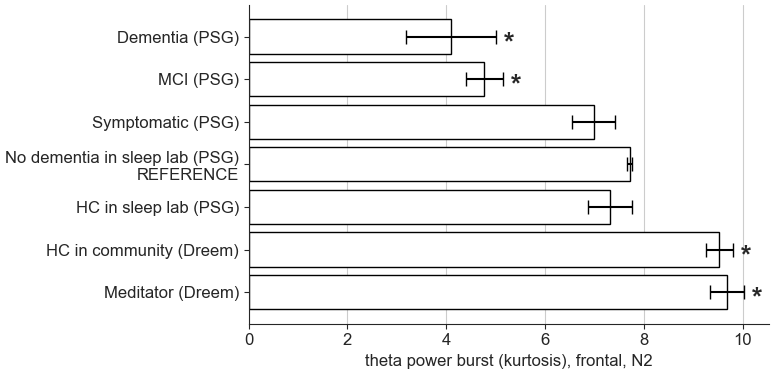


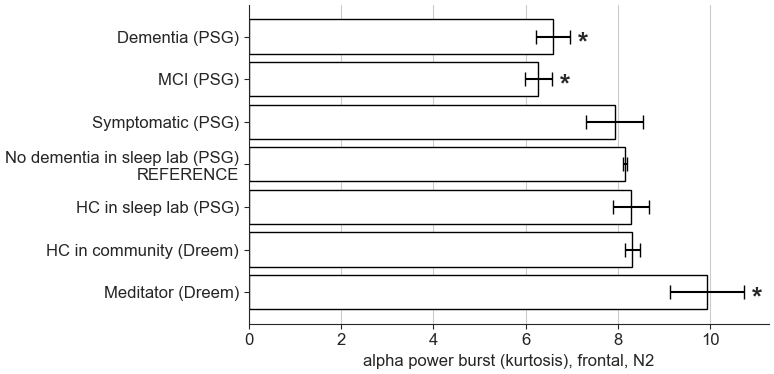


**Figure S3.** Top three EEG features common to both the brain age model trained on Dreem EEGs (frontal only) and the model trained on EEGs in the PSGs. (top left) Signal waveform high-amplitude burst, quantified by kurtosis of the amplitude distribution within each 30-second epoch, measured at the frontal channel during N2 stage. It is likely due to patterns such as K-complex. The statistical significance (*p <* 0.05) marked by * is obtained by comparing the matched cohort to the reference cohort (“No dementia in sleep lab (PSG)”) using a t-test. The error bar indicates the standard error. (top right) Theta power burst, quantified by kurtosis of the theta band power within each 30-second epoch measured at the frontal channel during N2 sleep. (bottom) Alpha power burst, quantified by kurtosis of the alpha band power within each 30-second epoch measured at the frontal channel during N2 sleep.

# Table S1. Unadjusted brain age index (BAI) by group

|  | **Meditatorᵃ (*n* = 34)** | **HC in communityᵃ (*n* = 1077)** | **HC in sleep labᵇ (*n* = 112)** | **No DEM in sleep labᵇ (*n* = 7618)** | **SYMᵇ**  **(*n* = 697)** | **MCIᵇ**  **(*n* = 205)** | **DEMᵇ**  **(*n* = 153)** |
| --- | --- | --- | --- | --- | --- | --- | --- |
| No. (%) with data | 33 (97) | 1077 (100) | 112 (100) | 7618 (100) | 697 (100) | 205 (100) | 153 (100) |
| BAI, mean (*SEM*), yr | -6.78 (1.27) | -0.41 (0.19) | 1.34 (0.52) | 2.35 (0.07) | 2.62 (0.22) | 2.97 (0.38) | 3.59 (0.47) |
| Age, mean (*SEM*), yr | 37.88 (1.54) | 45.57 (0.42) | 39.07 (1.20) | 48.94 (0.18) | 64.11 (0.34) | 67.98 (0.65) | 70.29 (0.83) |
| No. (%) female | 12 (36) | 191 (18) | 62 (55) | 3843 (50) | 389 (56) | 87 (42) | 69 (45) |
| *p*-valueᶜ | *p*< 0.001 | *p* < 0.001 | *p* > 0.05 | N/Aᵈ | *p* > 0.05 | *p* > 0.05 | *p* < 0.01 |

Abbreviations: BAI, brain age index; *SEM*, standard error of the mean; HC, healthy control; PSG, polysomnography; DEM, dementia; SYM, symptomatic; MCI, mild cognitive impairment.

ᵃData recorded using the Dreem device

ᵇData recorded in the Massachusetts General Hospital Sleep Lab using polysomnography

ᶜ*P*-value for *t*-test against the reference group, No Dementia

ᵈValue cannot be calculated for the reference group, No Dementia

# Table S2. Time comparison of NIH toolbox scores for the meditation cohort

| **Assessment**ᵃ | Pre-retreat raw score, mean (*SD*), median (*IQR*)ᵇ  (*n* = 35) | Post-retreat raw score, mean (*SD*), median (*IQR*)ᶜ  (*n* = 34) | Pre-retreat adjusted score, mean (*SD*), median (*IQR*)ᵇ  (*n* = 35) | Post-retreat adjusted score, mean (*SD*), median (*IQR*)ᶜ  (*n* = 34) |
| --- | --- | --- | --- | --- |
| **Cognitive scores** | | | | |
| Crystallized Comp.ᵈ | 111.353 (7.19), 111 (11.5) | 110.235 (6.601), 111 (7.75) | 56.029 (8.233), 56.5 (10.5) | 53.765 (8.995), 54 (11.75) |
| LSWMT | 19.647 (2.751), 19 (3.75) | 20.5 (3.387), 21 (5) | 53.941 (9.079), 52 (13) | 57.088 (12.124), 59.5 (18.75) |
| ORRT | 118.824 (7.586), 120 (5) | 115.029 (6.948), 116 (10) | 67.029 (9.292), 69.5 (11) | 61.412 (11.709), 60 (16.25) |
| PSMT | 18.412 (7.656), 19 (10) | 22.206 (7.623), 22.5 (13.25) | 57.912 (10.978), 56 (12.5) | 62.412 (12.572), 59 (21) |
| PVT | 103.294 (8.48), 102.5 (11.5) | 104.794 (8.559), 104 (12.5) | 43.971 (9.369), 41 (13.75) | 45.412 (8.978), 43 (14.5) |
| **Emotional scores** | | | | |
| Emotional Support | 30.588 (7.75), 32 (11.25) | 32.765 (6.569), 34 (9.75) | 43.676 (10.453), 44 (15.25) | 46.735 (10.652), 46 (15.5) |
| Friendship | 27.824 (6.658), 28 (7.75) | 29.647 (6.218), 29 (5.75) | 44.912 (8.607), 44.5 (11) | 47.794 (9.66), 46 (7.5) |
| Life Satisfaction | 26.588 (13.198), 23 (8.5) | 29.353 (13.547), 23 (7.5) | 55.676 (10.342), 55 (9.75) | 58.853 (8.55), 57 (7.5) |
| Instrumental Support | 31.765 (7.05), 32.5 (9.5) | 33.294 (5.452), 33.5 (5.5) | 48.735 (9.212), 48 (10.75) | 50.147 (7.993), 48.5 (8.5) |
| Loneliness | 9.147 (3.807), 8.5 (6.5) | 8.088 (2.734), 7.5 (5) | 50.324 (10.289), 50 (18.75) | 48.088 (8.554), 48.5 (17) |
| Perceived Stress | 21.794 (6.285), 23 (9.5) | 19.382 (5.416), 20.5 (8.25) | 45.765 (10.649), 48 (13.75) | 41.735 (9.922), 44 (13) |
| Positive Affect | 35.765 (22.349), 27 (40.5) | 29.676 (19.776), 16.5 (40.5) | 49.265 (8.099), 50 (9.25) | 55.824 (9.376), 51 (14.25) |

Abbreviations: NIH, National Institutes of Health; *IQR*, interquartile range; Crystallized Comp., crystallized cognition composite score; LSWMT, list sorting working memory test; ORRT, oral reading recognition test; PSMT, picture sequence memory test; PVT, picture vocabulary test

ᵃData reported as mean (*SD*), and median (*IQR*) for continuous variables.

ᵇData reported for all enrolled participants (*n* = 35) for the first assessment

ᶜData reported for 34 (97%) participants for the second assessment.

ᵈCrystallized composite score is a calculation provided by the NIH Toolbox that summarizes crystallized intelligence test scores. 3 fluid intelligence tests were omitted as they could not be completed over Zoom, so no composite fluid intelligence score was generated.

# Supplemental Analysis 1. Analysis of NIH toolbox (time comparison) using JASP software

The four cognitive tasks that we tested, the Picture Vocabulary Test (PVT), Oral Reading Recall Test (ORRT), List Sort Working Memory Task (LSWMT), and Picture Sequence Memory Test (PSMT) test both crystallized (PVT and ORRT) and fluid (LSWMT and PSMT) cognitive abilities.  The overall repeated measures ANOVA with the factor of Time (2 levels: Pre and Post meditation retreat) and Test Type (4 levels: PVT, ORRT, LSWMT, and PSMT) revealed no significant effect of Time (*F*_1,33_=0.6, *p* > 0.05), a significant effect of Test Type (*F*_3,99_=41.3, *p* < 0.001), and no significant Time x Test type interaction (*F*_3,99_=5.1, 0.001 < *p* < 0.05).

A separate repeated-measures ANOVA on the fluid cognition tasks (LSWMT and PSMT) revealed a non-significant but trend-level main effect of Time (*F*_1,33_=5.4, *p* < 0.05). The Test Type factor was non-significant but trend-level (*F*_1,33_=9.1, *p* < 0.05). The Time x Test type interaction was not significant (*F*_1,33_=0.2, *p* > 0.05). The changes in means and standard deviations Pre and Post meditation retreat are reported in Supplemental Table 2.

We analyzed the positive and negative affective measures separately, as increases in the positive affective measures indicate improvement, while increases in the negative measures indicate a decline in emotional well-being. We submitted the positive Affective measure scores to a repeated-measures ANOVA, with Test Type (5 levels: Positive Affect, Life Satisfaction, Emotional Support, Instrumental Support, Friendship and Time (2 levels: Pre and Post meditation intervention) as the factors. The Time factor was significant (*F*_1,33_=14.4, *p* < 0.001), indicating that positive affective measures changed between pre- and post-meditation retreat. The Test Type factor was also significant (*F*_4,132_=17.9, *p* < 0.001), indicating differential effects on the five positive affective measures that we tested.

For the negative measures of affect, Perceived Stress and Loneliness, the Time (Pre and Post meditation intervention) factor was non-significant but trend-level (*F*_1,33_= 8.8, *p* < 0.01), as was the Test type factor (*F*_1,33_=22.4, *p* < 0.001), indicating different outcomes on these measures. The Time x Test type interaction was not significant (*F*_1,33_=0.8, *p* > 0.05).

# Supplemental Method. Using linear discriminant analysis (LDA) to remove artifacts

We used LDA on EEG signals collected from the Dreem devices. The signals were segmented into 30-second epochs. The inputs to LDA are the total power and 2nd order difference (for abrupt non-physiological changes) of the spectrum of each 30-second epoch. To train the LDA classifier, we manually labeled each epoch for a selected set of EEGs with different ratios of definite artifact per epoch across the night: 10 EEGs with a ratio of 25-50%; and 10 EEGs with a ratio >50%. After training, the LDA classifier labeled each 30-second epoch as artifact or no artifact. By comparing these with manually assigned labels, we derived a receiver operating characteristic (ROC) curve. We selected an operating point for rejecting artifactual epochs by maximizing the Youden Index, which yielded a specificity of 86% and sensitivity of 84%. Visual inspection of this operating point confirmed it to be an acceptable tradeoff between retaining high-quality signals and rejecting artifacts. The LDA was run on both F7-O1 and F8-O2 channels. Epochs with artifact either in F7-O1 or F8-O2 are regarded as epochs with artifact.

# Supplemental Method. Definitions of dementia, MCI, symptomatic, no dementia, and healthy groups from the sleep lab

The participant groups that underwent overnight PSG at MGH were categorized into dementia, MCI, symptomatic, nondementia, and healthy groups. They were determined by natural language processing applied to the electronic medical record using custom software. Notably, in MGH electronic medical records, problem lists are lists of active, reoccurring medical problems for each patient, whereas encounter diagnoses are often preliminary diagnoses that physicians code for each patient encounter. To ensure the accuracy of our dementia and MCI groups, we used the problem list as a criterion because it is a more reliable source of diagnosis than only encounter diagnoses.

The inclusion criteria are based on data entered in the medical record before the sleep study or at most 1 year after the sleep study unless otherwise stated. A patient was in a group if they met at least 1 of the inclusion criteria and did not meet any of the exclusion criteria. All groups except the healthy group were mutually exclusive.

The definition of the dementia group is:

- Using >=1 dementia-related medication with a diagnosis containing any dementia keyword
- Diagnosis in the problem list containing any dementia keywords
- MoCA score <=19 and no MoCA scores >27 after the sleep study
- MMSE score <=25

Note:
Dementia-related medications include aricept, donepezil, exelon, rivastigmine, memantine, namenda, namzaric, razadyne, and galantamine.
Dementia keywords were dementia and Alzheimer’s.

The definition of the MCI group is:

- Diagnosis in the problem list containing any MCI keyword
- MoCA score 20-25 and no MoCA scores >27 after the sleep study

Note:
MCI keywords were MCI, mild cognitive impairment, and minimal cognitive impairment.

The definition of the symptomatic group is:

- Diagnosis containing any dementia or dementia-related keyword in the encounter diagnosis, problem list, and/or medical history

Note:
Dementia-related keywords were cognitive, memory, amnesia, agnosia, apraxia, aphasia, and mental.
We used encounter diagnosis as a criterion for the symptomatic group to make the symptomatic group inclusive of any patients with potential cognitive concerns and the nondementia group exclusively asymptomatic (i.e., the nondementia group was obtained by excluding the aforementioned groups).

The definition of the no dementia group is:

- Does not belong to dementia, MCI, or symptomatic group but may have a prior history of neurological or psychiatric disease in an encounter diagnosis, problem list, and/or medical history

Note:
Neurological or psychiatric diseases included cerebral hemorrhage, Parkinsonism, mood disorder, psychotic disorder, intracranial hypertension, cerebral palsy, epilepsy, hydrocephalus, encephalopathy, multiple sclerosis, and delirium.

The definition of the healthy control group is:

- Subset of the no dementia group with no history of neurological or psychiatric disease in encounter diagnosis, problem list, and/or medical history

Note:
Neurological or psychiatric diseases included cerebral hemorrhage, Parkinsonism, mood disorder, psychotic disorder, intracranial hypertension, cerebral palsy, epilepsy, hydrocephalus, encephalopathy, multiple sclerosis, and delirium.
